# Re-evaluation of the morphology and phylogeny of *Diplocynodon levantinicum* Huene & Nikoloff, 1963 and the stratigraphic age of the West Maritsa coal field (Upper Thrace Basin, Bulgaria)

**DOI:** 10.7717/peerj.14167

**Published:** 2022-11-09

**Authors:** Tobias Massonne, Madelaine Böhme

**Affiliations:** 1 Senckenberg Center of Human Evolution and Palaeoecology, Tübingen, Germany; 2 Department of Geosciences, Eberhard-Karls-Universität Tübingen, Tübingen, Germany

**Keywords:** Oligocene, Crocodylia, Biogeography, Diplocynodontinae, Bulgaria

## Abstract

*Diplocynodon levantinicum* Huene & Nikoloff, 1963 was described based on few bone fragments from the West-Maritsa lignite basin of Central Bulgaria. Huene & Nikoloff, 1963 assumed a late Pliocene age, implying that this species represents the stratigraphically youngest crocodilian of Europe. In this current study, we re-evaluate the stratigraphy of the West-Maritsa Basin and conclude a late Oligocene age of ~26 Ma for the Kipra coal-seam, the fossiliferous horizon. Furthermore, topotypical and undescribed *D. levantinicum* specimens are accessible now and allowed for a deeper taxonomic and phylogenetic analysis. A comparison with other *Diplocynodon* species reveals *D. levantinicum* as a valid species, having (1) a long suborbital fenestra, (2) a very short dentary symphysis, (3) a large gap between the first and second dentary alveolus, (4) an occlusion pit in line with the tooth row posterior to the 14^th^ dentary alveolus, (5) a sulcus lateral to the glenoid fossa and, (6) a lingual foramen for the articular artery situated entirely on the surangular. The phylogenetic analyses find *D. levantinicum* deeply nested inside the Diplocynodontinae subfamily. After the disappearance of the Paratethyan influence (Solenovian regional stage) in the Upper Thrace Basin this species has roamed during the late Oligocene extensive freshwater lake and swamp ecosystems represented by the Maritsa Formation. *Diplocynodon levantinicum* represents the only nominal *Diplocynodon* taxon of late Oligocene (Chattian) age.

## Introduction

*Diplocynodon* Pomel, 1848 is a genus of crocodilians exclusively known from European localities spanning nearly the whole Cenozoic from the upper Palaeocene to the end of the Middle Miocene Climatic Optimum (*e.g.*, Buscalioni, Sanz & Casanovas, 1992; Brochu, 1999; Böhme, 2003; Delfino, Böhme & Rook, 2007; Delfino & Smith, 2012; Rio et al., 2020). *Diplocynodon* fossils have been found in over 300 localities (Böhme & Ilg, 2003); however, surprisingly few occurrences (~10) are documented from the late Oligocene (Chattian), and all of them are from Western Europe (France, Switzerland, Germany). These Chattian specimens comprise mostly undiagnostic material (*e.g.*, teeth and osteoderms) and do not allow a species determination. From the Rupelian of France and Italy, *Diplocynodon* remains which were referred to *Diplocynodon ratelii* Pomel, 1847 (Brinkmann & Rauhe, 1998; Kotsakis, Delfino & Piras, 2004; Pandolfi et al., 2016) are known, but their affinity to this species is still discussed (Martin & Gross, 2011; Rio et al., 2020).

Until recently, a total of nine *Diplocynodon* taxa were considered valid (Luján et al., 2019; Rio et al., 2020; Chroust et al., 2021). More material from putative further taxa is known, but either poorly preserved or in need of revision like *Diplocynodon gervaisi* (Gervais, 1859) or *Diplocynodon buetikonensis* (Meyer, 1854) (Scherer, 1978; Scherer, 1979; Piras & Buscalioni, 2006; Scheyer, Straehl & Sánchez-Villagra, 2015). During the last decade, scientific studies focused primarily on Western and Central European material (*e.g.*, Martin & Gross, 2011; Martin et al., 2014; Pandolfi et al., 2016; Díaz Aráez et al., 2017; Chroust, Mazuch & Luján, 2019; Chroust et al., 2021), whereas Eastern Europe fell short. In the last 2 years, however, description of *Diplocynodon kochi* Venczel & Codrea, 2022 and further fragmentary material of Ukrainian and Romanian deposits reached attention (Codrea & Venczel, 2020; Kuzmin & Zvonok, 2021; Sabău et al., 2021; Venczel & Codrea, 2022). Reports of putative *Diplocynodon* remains of the late Cretaceous of Central Asia (Kuzmin & Zvonok, 2021) question the solely European radiation of *Diplocynodon*. *Diplocynodon* material from the late Cretaceous could also potentially fill the time gap between the upper Palaeocene remains of *Diplocynodon* from Western Europe and the putative age of the taxon indicated by its basal alligatoroid affinities on the phylogenetic tree (*e.g.*, Brochu, 1999; Martin, 2010; Martin et al., 2014; Rio et al., 2020; Massonne et al., 2019; Rio & Mannion, 2021).

Over 50 years ago, Huene & Nikoloff (1963) described fragmentary crocodilian material from the West-Maritsa lignite basin of Central Bulgaria and erected a new species: *Diplocynodon levantinicum*. The authors affiliated the material with *Diplocynodon* based on the confluent third and fourth dentary tooth, but did not compare the material with any other *Diplocynodon* species and did not offer any arguments for erecting a new species. The age of the West-Maritsa lignite basin was believed at this time to be of late Pliocene (Levantinian) age, characterizing it as the youngest fossil crocodilian from Europe (Huene & Nikoloff, 1963), which has been challenged several times (Böhme, 2003; Delfino & Rossi, 2013).

In the current study, we examined more material of *D. levantinicum* and compared it with all known *Diplocynodon* taxa and conducted phylogenetic analyses, which confirm that *D. levantinicum* represents indeed a valid species. We further re-examined the age of the West-Maritsa lignite basin and conclude a depositional age of ~26 Ma during the late Oligocene.

## Geological settings

**Figure 1:**
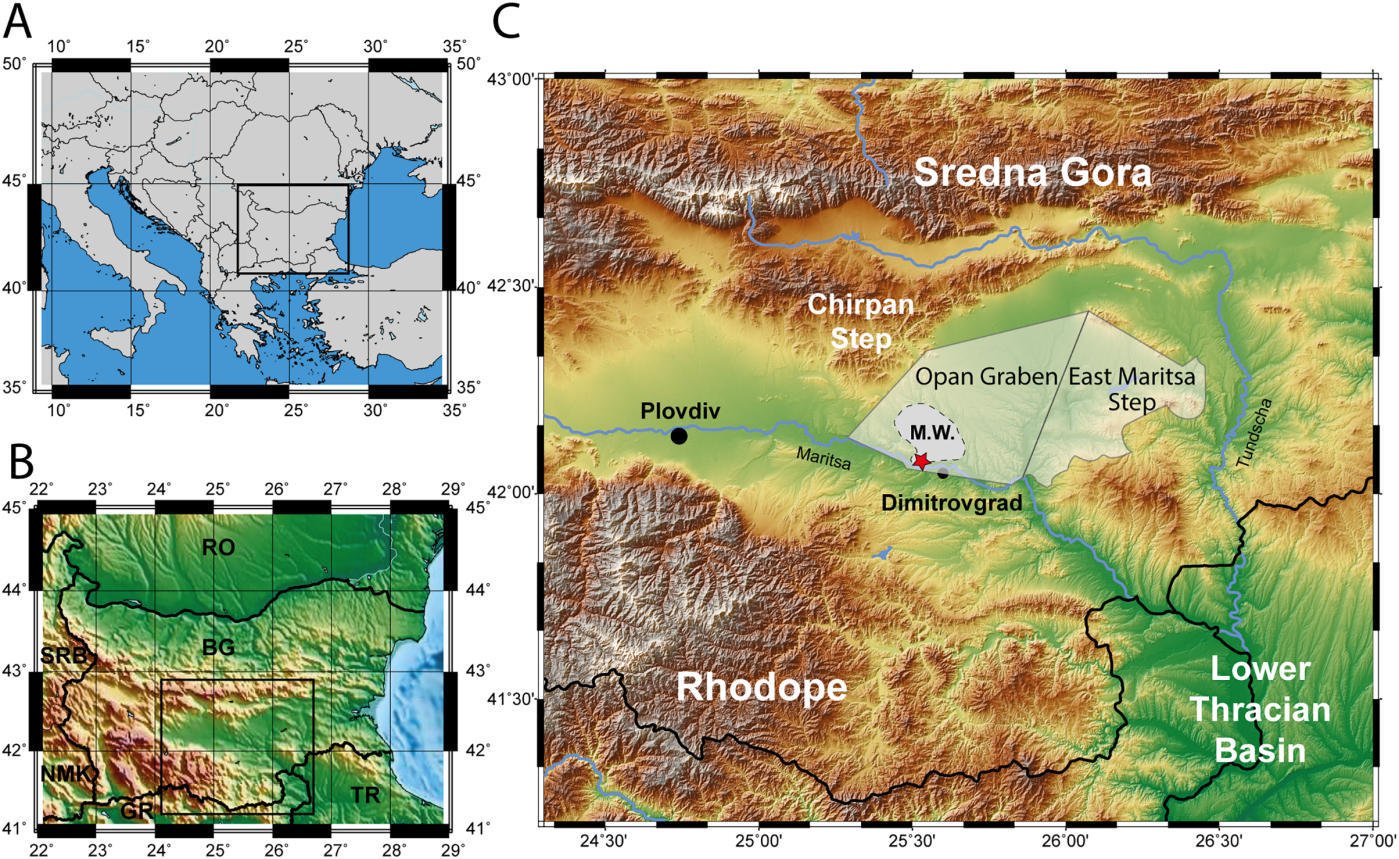
Map of the excavation site. (A) Map of South-eastern Europe. (B) Map of Bulgaria. (C) Topographic map of the Upper Thracian Basin in Bulgaria, between the Sredna Gora mountain in the north and the Rhodope mountain in the south. The eastern part of this basin, east of the Chirpan Step, is highlighted transparent with the deep Opan Graben and the shallow East Maritsa Step (after Popov, Velichkov & Popov, 2015, see text for further details). The extent of the West Maritsa coal field in the Orpan Graben is marked as M.W. The red star represents the position of the former Nadeshda and Radievo 1 underground coal-mines in the northwestern edge of present-day city of Dimitrovgrad. The maps have been created using the Generic Mapping Tools program (Wessel et al., 2019).

**Figure 2:**
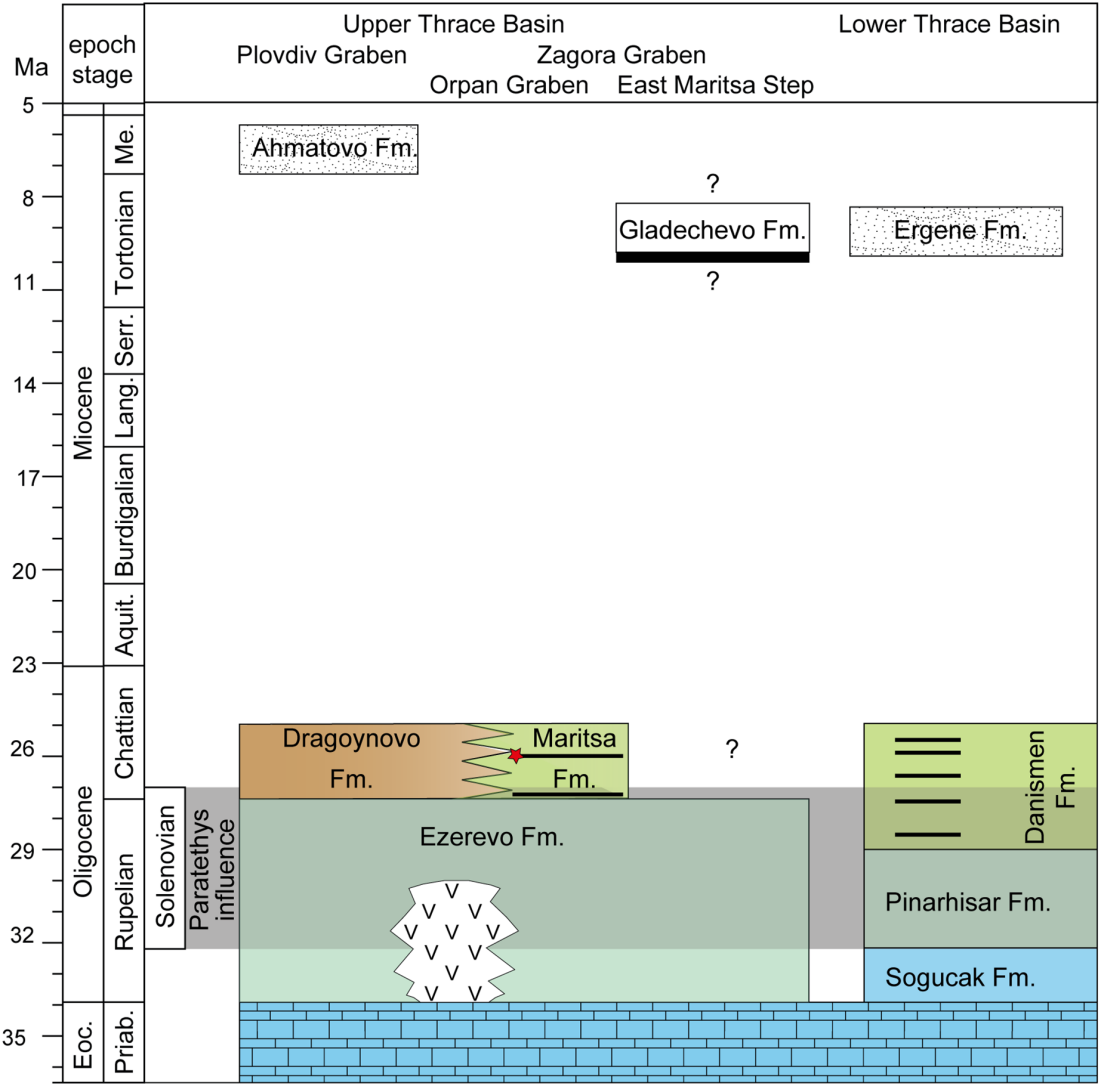
Stratigraphy of the upper and lower thrace basin. The stratigraphy of different parts in the Upper Thrace Basin (Bulgaria) is discussed in the text. The stratigraphic data from the Lower Thrace Basin (Turkey) are from İslamoğlu et al., 2008; Harzhauser et al., 2016. Blue colours denote to normal marine Tethys facies, blue-green colours to Paratethyan brackish waters (Ezerevo and Pinarhisar Formation), and green and brown colours represent lacustrine, respectively continental facies. Black stripes designate coal-seams, the red star symbolize the stratigraphic position of the fossil locality in the Kipra coal-seam, and the period of Paratethyan influence during the Solenovian is highlighted in grey. White colours represent fluvial formations during the late Miocene.

**Geologic and stratigraphic overview on the West Maritsa coal field:** The Upper Thracian Basin represents a complex post-collisional rift system divided into several tectonic units, the larger ones represented by the Plovdiv and the Zagora Grabens, divided by the Chirpan step (Popov, Velichkov & Popov, 2015). The eastern part of this rift system, the Zagora Graben, is further structurally subdivided into several steps and grabens, as the East Maritsa Step and the Opan Graben, to which the West Maritsa coal field belongs (Figs. 1 and 2). The ~2 km deep Opan Graben (Krâstev, Dobrev & Dragomanov, 1992) is filled with Middle Eocene to Early Oligocene sediments, as *e.g.*, marine carbonates, brackish-to-freshwater siltstones and marls and terrigenous siliciclastics and pyroclastics, followed by Neogene and Quaternary terrestrial deposits (Bojanov et al., 1993). The Oligocene sediments below the West Maritsa coal field belong to the Ezerovo Formation (Kojumdgieva & Dragomanov, 1979), which is here 150–170 m thick (Kamenov & Panov, 1976). The Ezerevo Formation contains a brackish water Paratethyan mollusc fauna of the regional Solenovian stage, which corresponds to the middle and late Rupelian (Kojumdgieva & Sapundgieva, 1981). At the top of the Ezerovo Formation, the Paratethyan endemics disappear and are replaced by a non-Paratethyan fresh-to-brackish water mollusc association with *Polymesoda subarata convexa, Melanopsis hantkeni, Vitta rumeliana*. This transition is placed near the Rupelian-Chattian (Solenovian-Kalmykian) boundary (Kojumdgieva & Sapundgieva, 1981; Popov & Studencka, 2015) and is coeval to the brackish-to-freshwater environmental transition involving very similar mollusc assemblages in the nearby Lower Thrace Basin of Turkey (İslamoğlu et al., 2008; Harzhauser et al., 2016). The Ezerovo Formation is concordantly overlaid by the over 500 m thick coal-bearing Maritsa Formation (Panov, 1962; Kojumdgieva & Dragomanov, 1979). Its base is defined by the lowest coal seam, but the environmental changes are transitional, as evidenced by the persistence of the *Polymesoda*-association into the lowest parts of the Maritsa Formation (Kojumdgieva & Dragomanov, 1979), in addition to a Rupelian brackish water fish species, *Dapalis macrurus*, found between the first and second coal seam (= Maritsa seam) and described by Brunkin, Obrhelova & Dimitrov (1983). Therefore, we conclude that the base of the Maritsa Formation in the Opan Graben correlates to the Rupelian-Chattian transition.

Further evidence for an Oligocene age can be provided with a fossil leaf-assemblage from sandstones near Merichleri on the western border of the Opan Graben, belonging to the lower part of the Maritsa Formation (Коnyarov, 1932: fig. 43). Коnyarov (1932) determined and figured (his pl. 64-66), among others, *Eotrigonobalanus furcinervis* and *Sabal* palms. The evergreen oak *E. furcinervis* is a common and widespread tree during the middle Eocene to Oligocene of Europe (Mai, 1995), and is well-known from the Lower Thrace Basin and the Eastern Rhodopes from early Oligocene sediments and pyroclastics by leaves and silicified wood (Velitzelos, Kacek & Walter, 1999; Iamandei et al., 2013).

As evidenced by the labels, the fossils of *D. levantinicum* are found in the so called Kipra coal-seam of the underground mines Nadeshda (as the holotype of Huene & Nikoloff, 1963) and Radievo 1. The Nadeshda mine is situated 4.5 km NNE of the city centre of Dimitrovgrad, on the southern rim of the Opan Graben (see Коnyarov, 1932: fig. 43), and the Radievo 1 mine about 3 km further east. The Kipra coal seam is the third seam in the Maritsa Formation in the Opan Graben (counted from the base of the formation; 1–first seam, 2–Maritsa seam, 3–Kipra seam, 4–Havuzki seam (Коnyarov, 1932). The sedimentation between the seams is rather uniform and consists of greenish clays, silts and rarely marls, which frequently contain a pulmonate (*Planorbarius*, *Planorbis*) and terrestrial (*Helix*) gastropod fauna (Коnyarov, 1932), indicating a freshwater, lacustrine environment, interrupted by swampy conditions during lignite formation. The first two seams are spaced only 3 m apart (Brunkin, 1986) at the base of the formation, but the stratigraphic distance between the Maritsa and Kipra seam is not properly known, but may account to more than 300 m (Kostova et al., 2005: fig. 2). The lack of hiatuses in this tectonically very active graben, and the continuous transition between the Ezerovo and Maritsa Formations, followed by lacustrine sedimentation are arguments in favour that the sedimentation of the Kipra seam still occurs during the Chattian (Late Oligocene). Assuming a sedimentation rate of 30–50 cm per kyr, the ~300 m of lacustrine sedimentation would not last over 1 myrs. We therefore assume an age of about 26 Ma (corresponding to Paleogene mammal unit MP26) for *D. levantinicum*.

**Evaluation of former arguments for a younger age:** The Maritsa Formation in the West Maritsa coal-field has been formerly regarded as Pliocene (“Levantinian”, which is an older term and today corresponds to the late Pliocene), which has made the *Diplocynodon* and the “*Dorcatherium*” from the Kipra seam by far the latest records of crocodiles and tragulids in Europe. The reasons for this assumption have been three-fold: (1) early investigators related surface (or near-surface) finds of large mammals outside the Zagora Graben to the age of the West Maritsa coal-field, (2) mis-identification of molluscs, and (3) a lithostratigraphic correlation of the coal-bearing formations from the West and East Maritsa coal-fields.

Ferdinand von Hochstetter (1869), the first investigator of the Geology of Bulgaria, regarded the Upper Thrace Basin as Quaternary in age. Theodor Fuchs (1879) reported, from sands just below late Pleistocene loess, an early Pleistocene association of *Mammuthus meridionalis* and *Hippopotamus major* from a village directly south of Nova Zagora (old name Jeni Saghra), which is outside the Zagora Graben. In the same year Anton Petz (1879) mentioned teeth of *Mammuthus* from the same locality as well as from loessic sediments directly south of Popovitsa (old name Papazly; Plovdiv district), located also outside the Zagora Graben.

Based on these data and the Pliocene mastodon taxa *Anancus arvernensis* and “*Mammut borsoni”* from Parvomay (old name Borisovgrad, Plovdiv district), Asenovgrad (old name Stanimaka, Plovdiv district), and Haskovo (all outside the Zagora Graben), Zlatarski (1927 p. 213) interpreted the age of the “Maritsa Basin” as late Pliocene (Levantian).

Коnyarov (1932) agreed with Zlatarski’s conclusion and added a surface find of *Equus cabalus fossilis* from Merichleri (western rim of the Opan Graben). Specifically, he correlated the West Maritsa coal field with the Neogene succession of the Sofia Basin (assumed by him to be of “Levantian” age), by reporting shared pulmonate gastropod species with the Maritsa Formation and findings of *Dreissena bulgarica* from a drill-core (southern rim of the Opan Graben) at over 300 m depth near the base of the Maritsa Formation. However, according to Kojumdgieva & Sapundgieva (1981), this specimen belongs to *Andrusoviconcha euchroma*, typical for their Solenovian *Polymesoda*-association.

Finally, Brunkin (1986) correlated the Maritsa Formation from the deep Opan Graben (West Maritsa coal-field), with the Miocene, coal-bearing succession on the shallow East Maritsa Step (East Maritsa coal field). In East Maritsa, the productive main seam (Troyanovo seam in Troyanovo open-cast mines) is up to 24 m thick and concordantly overlain by the Gladechevo Formation (Nedjalkov & Kojumdgieva, 1983). Afterwards, 39 m above the main seam (layer 19 according Nedjalkov & Kojumdgieva, 1983) Kovachev (2004) discovered a skull of the Turolian proboscidian *“Mastodon” grandincisivus*, indicating a Turolian age (Markov, 2008). This is further corroborated by our recent findings of *Choerolophodon*, *Hipparion*, and a large-size giraffid from the same layer and directly below (M. Böhme, 2015, unpublished data). Therefore, the latter confirms East Maritsa coal horizons are late Miocene in age, whereas the coal in the West Maritsa field is late Oligocene in age (Fig. 2). The term Maritsa Formation should only be applied for the West Maritsa field in the Opan Graben.

**Correlation between the Upper and Lower Thrace Basins:** The Upper Thrace Basin, therefore, shows near the Rupelian-Chattian boundary a concordant transition from Solenovian brackish waters with an Eastern Paratethys fauna (Ezerovo Formation) to continental sedimentation, represented by the lacustrine to palustrine Maritsa Formation in the Opan Graben, and by the fluvial to alluvial Dragoynovo Formation (Kojumdgieva & Dragomanov, 1979) in the Plovdiv Graben to the west (Fig. 2).

In the Lower Thrace Basin, which is very similar in facies evolution, fauna and stratigraphy (İslamoğlu et al., 2008; Harzhauser et al., 2016) to the Opan Graben succession in the Upper Thrace Basin, Late Oligocene coal seams contain quite frequently small anthracothere artiodactyls of the genus *Elomeryx* (Lebküchner, 1974). In fact, İslamoğlu et al. (2008) described *Elomeryx borbonicus*, as typical for MP26-27 from the Tozaklı mine. We therefore conclude that coal formations in the Upper and Lower Thrace Basins are largely synchronous between 27 and 25 Ma.

**The Kipra coal seam and its fauna:** According to Коnyarov (1932), the Kipra coal seam is in maximum 4.45 m thick and subdivided into four sub-seams by three marl horizons, each 20 to 40 cm thick. Only the lower two sub-seams have been commercially excavated, and the upper of the two was known for its richness in vertebrates, so that miners called this sub-seam “Shompal” (Коnyarov, 1932), which means “ramrod”.

Коnyarov (1932) mentioned abundant fish remains and turtles, determined as *Emys* (= *Promalacoclemmys*) cf. *laharpi* (Pictet & Humbert, 1861), a species known from the Late Oligocene of Rochette in Switzerland (Hervet, 2004). From the “various other vertebrates”, he observed (Коnyarov, 1932: pl. 68) a mandible determined as *Tapirus* cf. *helveticus*, but the specimen, which rather resembles a medium-sized anthracothere, seems to be lost (N. Spassov, 2022, personal communication). In 1962, Bakalov & Nikolov, 1962 described “*Dorcatherium*” *bulgaricum*, a mandible from the Kipra seam from mine Radievo. This specimen has never been revised, and several authors express their doubts that it belongs to *Dorcatherium*, a genus known only from the Miocene. According to its description and figures, it may instead belong to an Oligocene stem-tragulid (Morales, Sánchez & Quiralte, 2012; Aiglstorfer, Rössner & Böhme, 2014).

## Materials and Methods

All of the herein described crocodilian material was found in the Kipra coal-seam of the underground mines Nadeshda and Radievo 1, which belong to the upper Oligocene West-Maritsa Basin, north to the City of Dimitrovgrad and east from the City of Plovdiv, in Central Bulgaria. The material is stored in the collection of the National Museum of Natural History in Sofia and consists of multiple lower jaw fragments, three skull fragments, and postcranial material and belonged to at least six different individuals. Based on the close distance of the underground mines Nadeshda and Radievo 1, and identical stratigraphic age of the mined Kipra seam, as well as the lacking of distinguishable differences and general similar size ratio of the material, all bones were assigned to a single species. Some of the material was already briefly described by Huene & Nikoloff (1963) and was given the name *Diplocynodon levantinicum*.

For the phylogenetic analyses, we used the dataset of Rio et al. (2020) (see File S1), which is mainly based on the dataset of Martin et al. (2014); Brochu et al. (2012) and Brochu & Storrs (2012), and added two additional taxa, *D. kochi* and *D. levantinicum*. A total of 16 rescorings were made for five taxa (see File S2). The expanded dataset includes 187 characters and 105 taxa.

For the phylogenetic analyses multiple characters were treated as ordered (17, 40, 49, 55, 63, 82, 88, 148) as in Rio et al. (2020).

We performed three different analyses: (1) without molecular constraints and unweighted characters, (2) with unweighted characters and with molecular constraints, based on Oaks (2011), in which the morphological data places fossil taxa within the forced molecular topology, as in Walter et al. (2021), (3) without molecular constraints and extended implied weighting *k* = 20 to decrease the impact of variable characters, as in Rio et al. (2020) (for further information and manual see Goloboff, 2014).

For all setups, we conducted maximum parsimony analyses in TNT 1.5 standard version, updated on March 31, 2021 (Goloboff & Catalano, 2016). All characters were treated as equally weighted; the maximum of trees was set to 99,999 and the tree replications to 1,000. For swapping algorithm, we used tree bisection reconnection with 10 trees saved per replication. A first run of heuristic search tree-bisection-reconnection failed to find all the most parsimonious trees (MPT) and, therefore, the heuristic search was repeated until the MPTs were found 50 times during each replicate (using the command ‘xmult = hits 50;’), as in Massonne et al. (2019) and Massonne et al. (2021). The trees retained in the memory were exposed to a second round of tree-bisection-reconnection.

We further conducted New Technology Search analyses for all setups, due to the large dataset (Goloboff, Farris & Nixon, 2008). The random addition sequence was set to 1,000. As search algorithm, sect. search, ratchet and tree fusing were used. For sect. search in the RSS settings, the maximal sector size was set to 58 representing half of the taxa in the dataset, in the CSS settings the rounds were set to 100 and the minimal sector size to 5, and for the XSS settings the number of rounds was set to 10. In the ratchet settings, the total number of iterations was set to 100, for tree fusing the rounds were set to 100. All other options were left as default. After the first round, we conducted a second round of new technology search with the trees saved from ram. Sectoral search was disabled, and we changed the number of iterations in the ratchet settings to 1,000 and the tree fusing to 1,000 rounds. The result was filtered for suboptimal trees, and the analysis was run again until the number of found trees did not change anymore.

## Systematic palaeontology

**Eusuchia** Huxley, 1875, *sensu* Brochu, 2003

**Crocodylia** Gmelin, 1789, *sensu* Benton & Clark, 1988

**Alligatoroidea** Gray, 1844, *sensu* Brochu, 2003

**Diplocynodontinae** Brochu, 1999

***Diplocynodon*** Pomel, 1847

***Diplocynodon levantinicum*** Huene & Nikoloff, 1963

**Figure 3:**
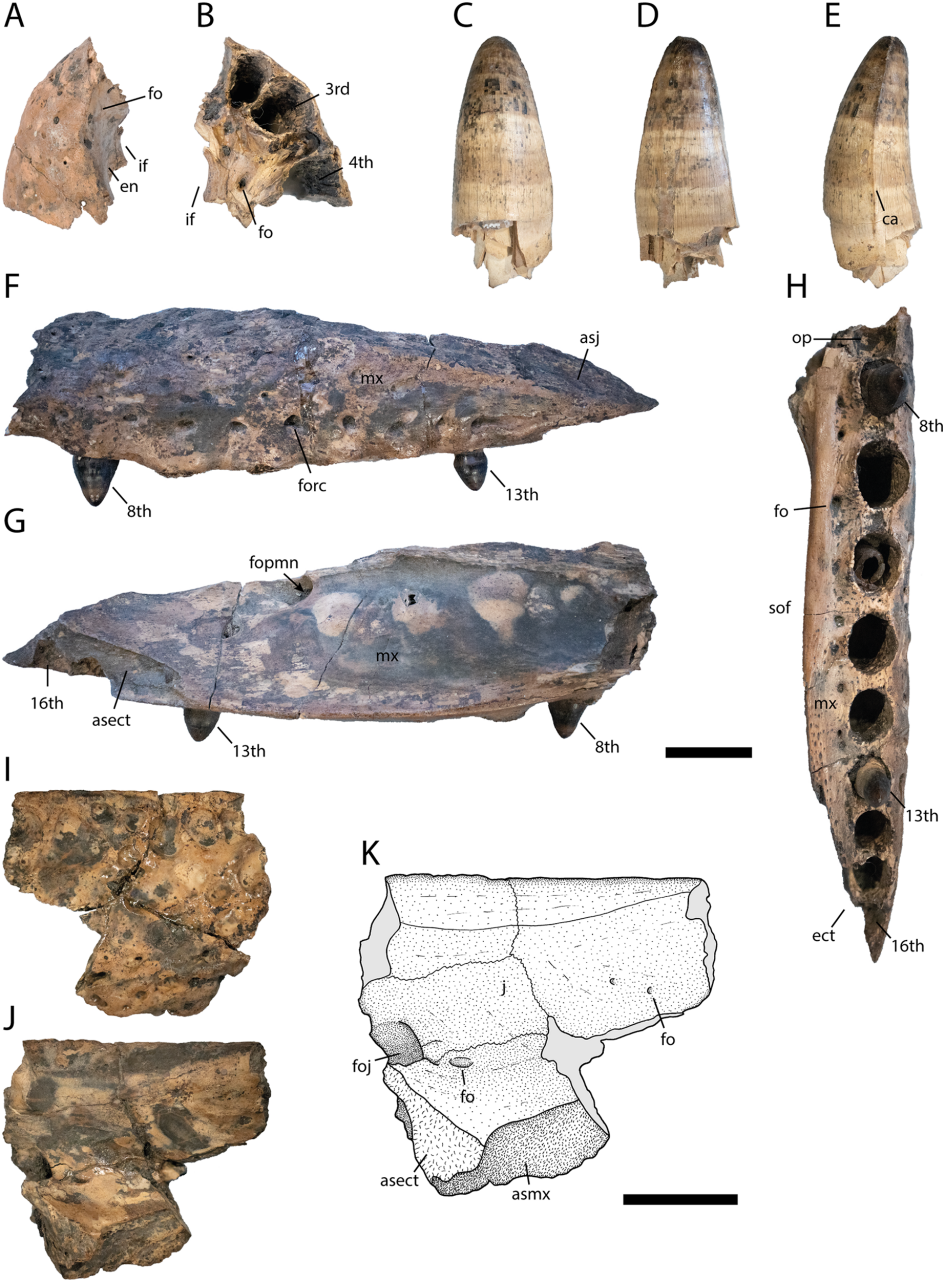
Skull elements of *Diplocynodon levantinicum* (NMNHS FR 30), underground coal mine close to the city of Dimitrovgrad, late Oligocene, Bulgaria. Premaxilla fragment (NMNHS FR 30-1) from Radievo 1 in (A) dorsal and (B) ventral view. Tooth (NMNHS FR 30-24) from Nadeshda in (C) lateral, (D) medial and (E) anteroposterior view. Maxilla fragment (NMNHS FR 30-21) from Nadeshda in (F) lateral, (G) medial and (H) ventral view. Jugal (NMNHS FR 30-22) from Nadeshda in (I) lateral and (J and K) medial view. Abbreviations: asect, articulation surface for the ectopterygoid; asj, articulation surface for the jugal; asmx, articulation surface for the maxilla; ca, carina; ect, ectopterygoid; en, external naris; fo, foramen; foj, foramen jugularis; fopmn, foramen for the posterior branch of the maxillary nerve; forc, foramen for the receptor canals; if, incisive foramen; j, jugal; mx, maxilla; op, occlusion pit; sof, suborbital fenestra. Scale = 1 cm.

**Figure 4:**
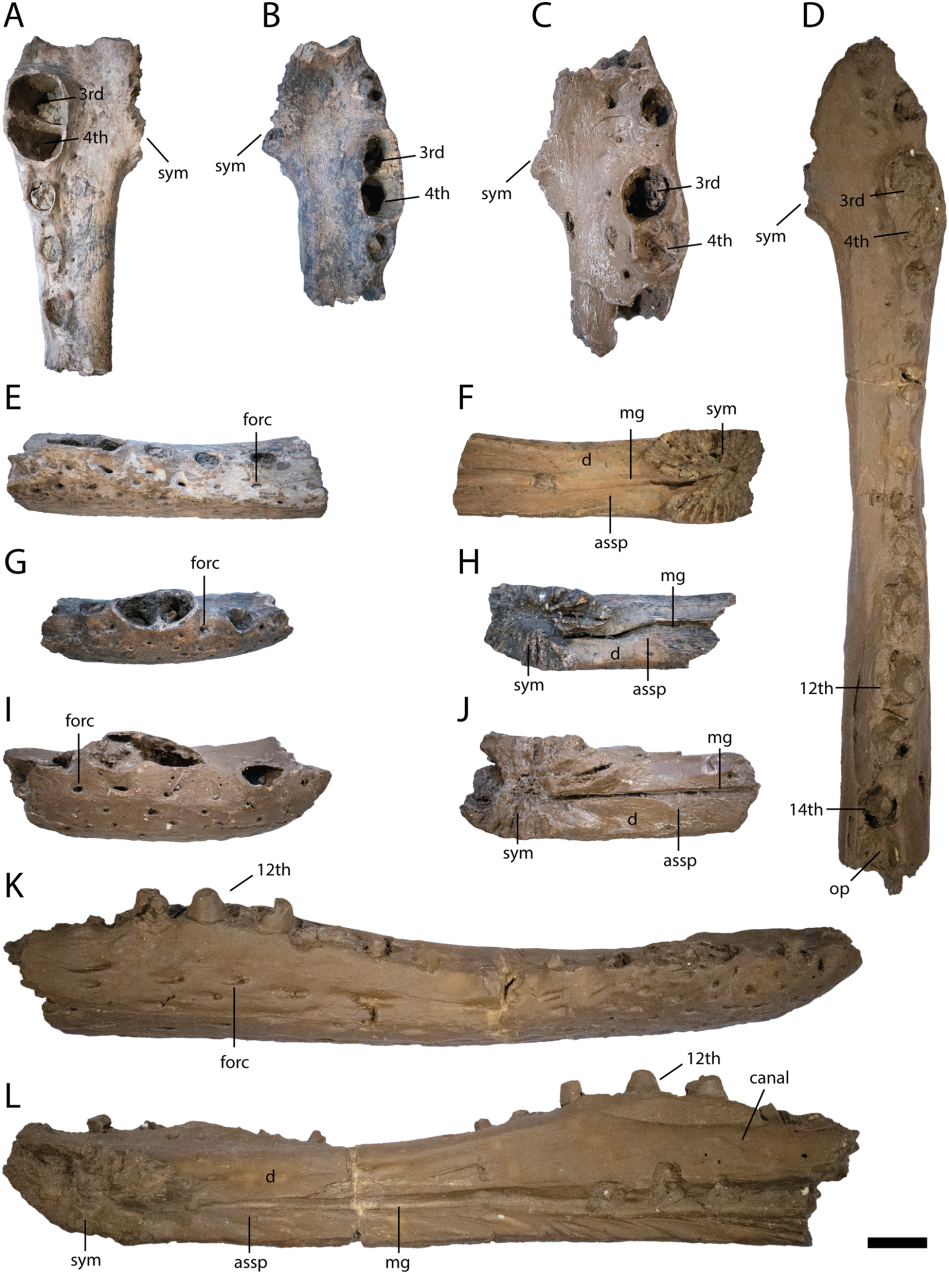
Anterior lower jaw elements of *Diplocynodon levantinicum* (NMNHS FR 30, NMNHS FR-31), underground coal mine close to the city of Dimitrovgrad, late Oligocene, Bulgaria. Dentary fragment (NMNHS FR 30-2) from Radievo 1 in (A) dorsal, (E) lateral and (F) medial view. Dentary fragment (NMNHS FR 30-23) from Radievo 1 in (B) dorsal, (G) lateral and (H) medial view. Dentary fragment (cast) (NMNHS FR 31-2) from Nadeshda in (C) dorsal, (I) lateral and (J) medial view. Dentary fragment (cast) (NMNHS FR 31-1) from Nadeshda in (D) dorsal, (K) lateral and (L) medial view. Abbreviations: assp, articulation surface for the splenial; d, dentary; mg, Meckelian groove; forc, foramen for the receptor canals; op, occlusion pit; sym, symphysis. Scale = 1 cm.

**Figure 5:**
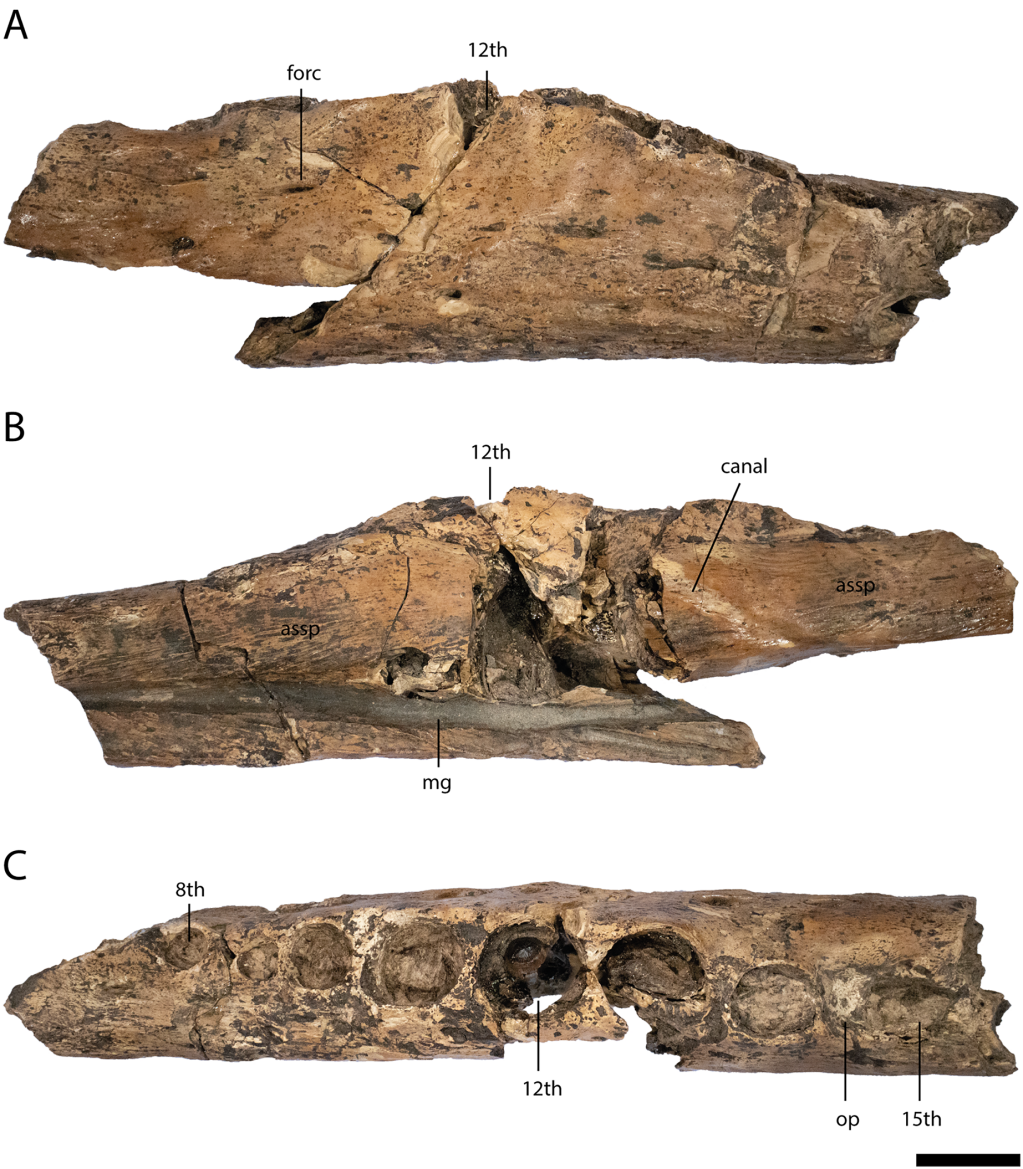
Lower jaw element of *Diplocynodon levantinicum* (NMNHS FR 30-3), underground coal mine close to the city of Dimitrovgrad, late Oligocene, Bulgaria. Lower jaw fragment (NMNHS FR 30-3) from Radievo 1 in (A) lateral, (B) medial and (C) dorsal view. Abbreviations: assp, articulation surface for the splenial; forc, foramen for the receptor canals; mg, Meckelian groove; op, occlusion pit. Scale = 1 cm.

**Figure 6:**
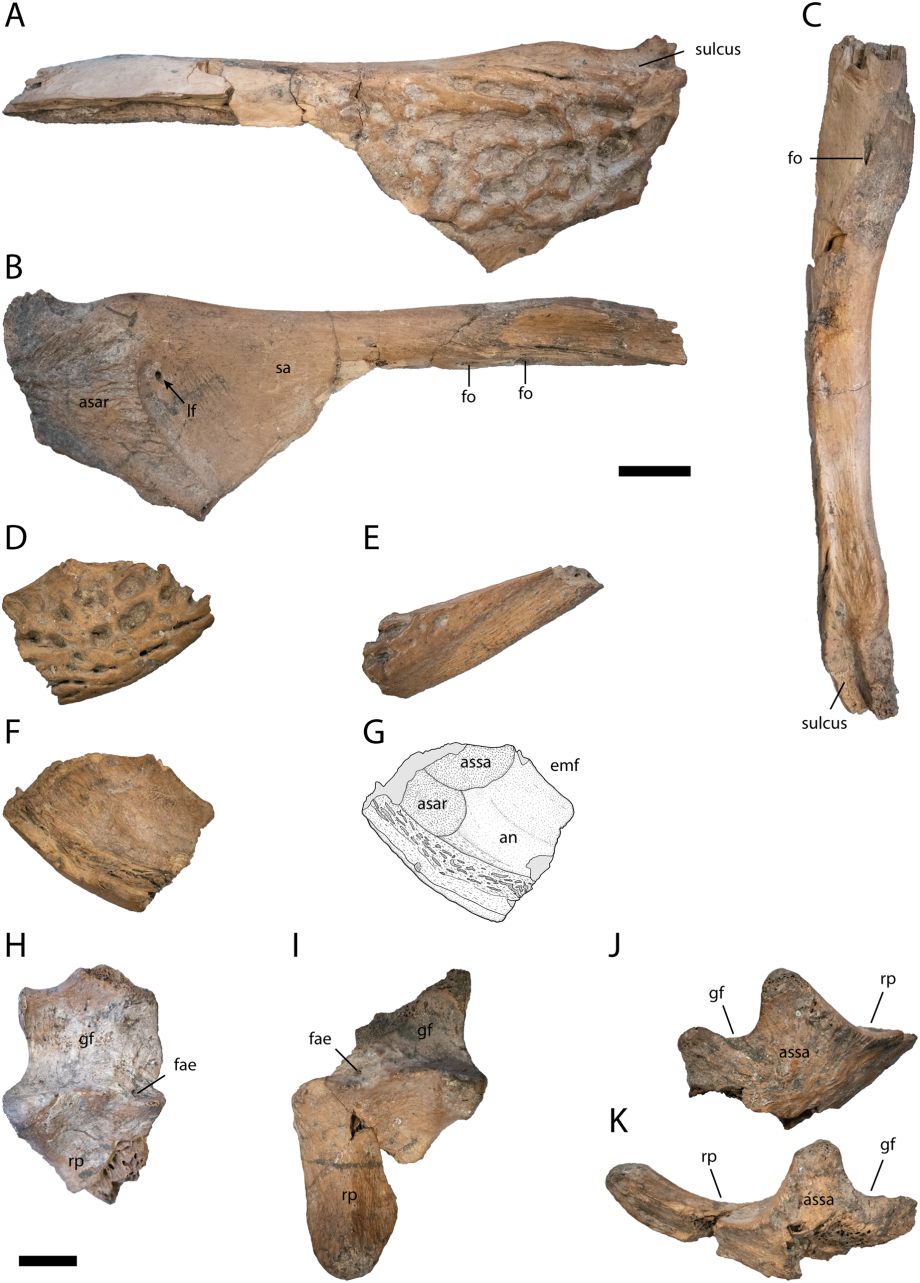
Posterior lower jaw elements of *Diplocynodon levantinicum* (NMNHS FR 30), underground coal mine close to the city of Dimitrovgrad, late Oligocene, Bulgaria. Surangular fragment (NMNHS FR 30-5) from Radievo 1 in (A) lateral, (B) medial and (C) dorsal view. Posteroventral angular fragment (NMNHS FR 30-6) from Radievo 1 in (D) lateral and (F and G) medial view. Posterior angular fragment (NMNHS FR 30-7) from Radievo 1 in (E) lateral view. Left articular (NMNHS FR 30-8) from Radievo 1 in (H) dorsal and (J) lateral view. Right articular (NMNHS FR 30-9) from Radievo 1 in (I) dorsal and (K) lateral view. Abbreviations: an, angular; asar, articulation surface for the articular; assa, articulation surface for the surangular; emf, external mandibular fenestra; fae, foramen aerum; fo, foramen; gf, glenoid fossa; lf, lingual foramen; rp, retroarticular process; sa, surangular. Scale = 1 cm.

**Figure 7:**
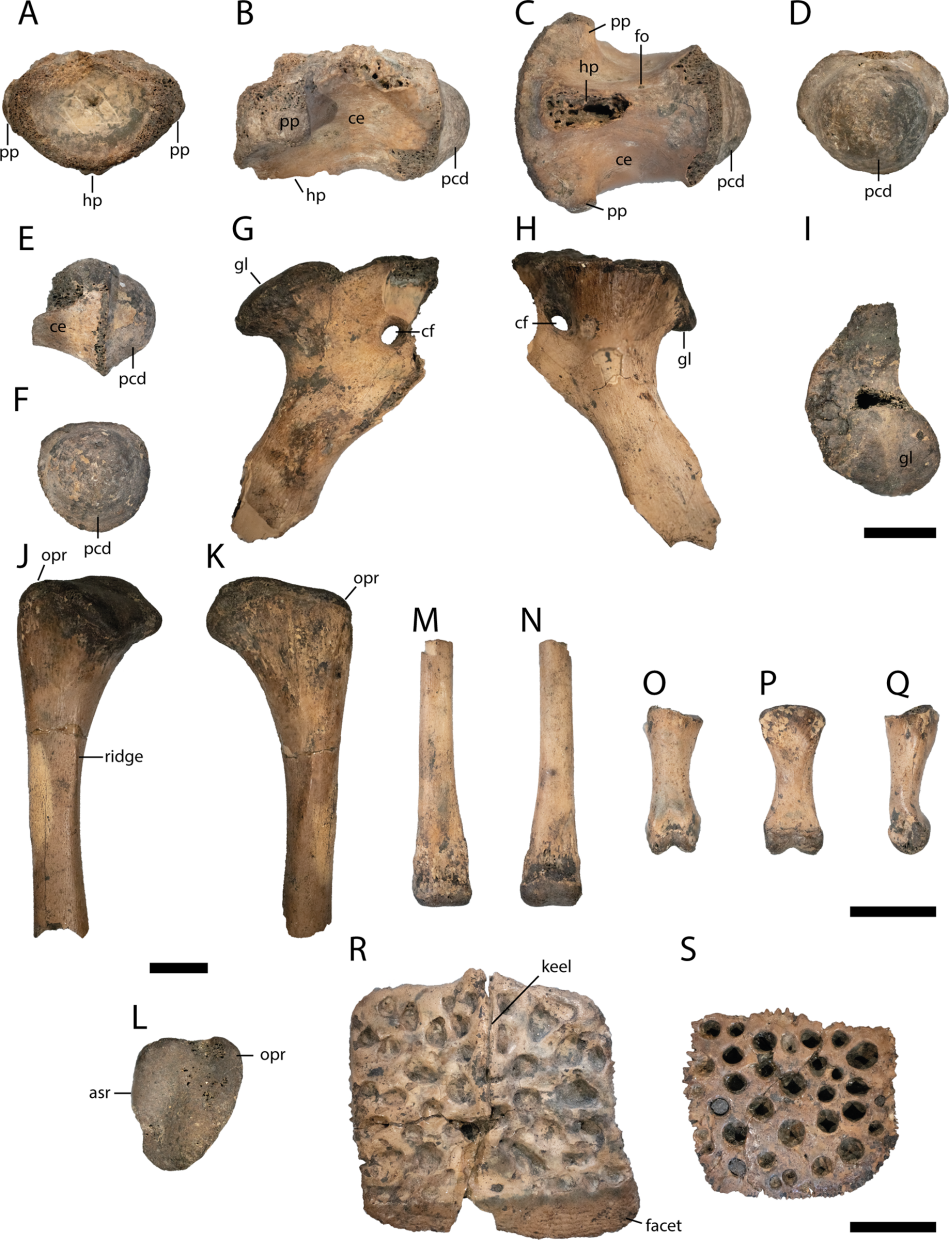
Postcranial elements of *Diplocynodon levantinicum* (NMNHS FR 30), underground coal mine close to the city of Dimitrovgrad, late Oligocene, Bulgaria. Cervical vertebra fragment (NMNHS FR 30-10) from Radievo 1 in (A) anterior, (B) lateral, (C) ventral and (D) posterior view. Caudal vertebra fragment (NMNHS FR 30-11) from Radievo 1 in (E) lateral and (F) posterior view. Right coracoid fragment (NMNHS FR 30-12) from Nadeshda in (G) lateral, (H) medial and (I) proximal view. Right ulna fragment (NMNHS FR 30-13) from Radievo 1 in (J) lateral, (K) medial and (L) proximal view, Metatarsal fragment (NMNHS FR 30-14) from Radievo 1 in (M) dorsal and (N) ventral view. Phalange (NMNHS FR 30-15) from Radievo 1 in (O) dorsal, (P) ventral and (Q) lateromedial view. Dorsal osteoderm (NMNHS FR 30-16) from Nadeshda in (R) dorsal view. Ventral osteoderm (NMNHS FR 30-19) from Nadeshda in (S) ventral view. Abbreviations: asr, articulation surface for the radius; ce, centrum; cf, coracoid foramen; gl, glenoid; hp, hypapophysis; opr, olecranon process; pcd, posterior condylus; pp, parapophysis. Scale = 1 cm.

**Genus diagnosis:** *Diplocynodon* is diagnosed by the combination of the following characters: (1) confluent dentary alveoli (shared with *Borealosuchus* spp., *Leidyosuchus canadensis* Lambe, 1907, *Deinosuchus riograndensis* Colbert, Bird & Brown, 1954 and *Bernissartia fagesii* Dollo, 1883), (2) bipartite ventral osteoderms (shared with *Borealosuchus* spp., extant *Caiman* spp. and *Tsoabichi greenriverensis* Brochu, 2010), (3) an axial hypapophysis close to the centre of the centrum (shared with some crocodyloids and orientalosuchines), (4) an ilium with a rounded dorsal margin and a very deep iliac posterior tip of the iliac blade (unknown for any other genus), (5) a linear frontoparietal suture between the supratemporal fenestrae (shared with multiple other eusuchians), (6) one row of postoccipital osteoderms (shared with *Alligator sinensis* Fauvel, 1879, *Paleosuchus palpebrosus* (Cuvier, 1807) and *Tomistoma schlegelii* (Müller, 1838), but in the majority of taxa not preserved) *Diplocynodon* Pomel, 1847 and *Diplocynodon levantinicum* Huene & Nikoloff, 1963 (Figs. 3–7).

**Diagnosis:** *Diplocynodon levantinicum* is a medium to large sized member of Diplocynodontinae, with a skull length of at least 250 mm (based on the largest lower jaw fragment), but separated teeth, likely associated with *D. levantinicum* (Figs. 3C–3E), indicate that individuals of 350–400 mm skull length were also present at the fossil site. Based on the skull to body length ratio of 1:7, the estimated total body length lied between 1.75 and 3.00 m (Whitaker & Whitaker, 2008). *Diplocynodon levantinicum* can be diagnosed by the combination of the following characters: having (1) a very short dentary symphysis reaching only the third to fourth dentary alveolus, (2) a large gap between the first and second dentary alveolus, (3) a lingual foramen for the articular artery situated entirely on the surangular, (4) a long suborbital fenestra reaching the level of the eighth maxillary alveolus, (5) a large sulcus next to the anterior half of the glenoid fossa, and (6) an occlusion pit in line with the tooth row posterior to the 14^th^ dentary alveolus.

*Diplocynodon levantinicum* can be differentiated from *Diplocynodon darwini* (Ludwig, 1877) in having (1) a dorsally projected external naris, (2) a large medial jugal foramen, (3) a shorter mandibular symphysis only reaching the level of the third to fourth alveolus, (4) a lingual foramen for the articular artery perforating only the surangular, and (5) an occlusion pit in line with the tooth row posterior to the 14^th^ dentary alveolus.

*Diplocynodon deponiae* (Frey, Laemmert & Rieß, 1987) in having (1) a narrow maxilla, not broader than the tooth row between the tooth row and the suborbital fenestra, (2) an ectopterygoid abutting the last maxillary alveolus, (3) a splenial excluded from the mandibular symphysis, (4) an occlusion pit in line with the tooth row posterior to the 14^th^ dentary alveolus, and (5) a large sulcus next to the anterior half of the glenoid fossa.

*Diplocynodon elavericus* Martin, 2010 in having (1) a narrow maxilla, not broader than the tooth row between the tooth row and the suborbital fenestra, (2) a linear maxillary tooth row posterior to the sixth maxillary alveolus, (3) a linear medial margin of the maxilla, (4) a shorter mandibular symphysis only reaching the level of the third to fourth dentary alveolus, and (5) posterior teeth and alveoli circular in cross-section.

*Diplocynodon hantoniensis* (Wood, 1846) in having (1) a smooth premaxilla surface lateral to the naris, (2) a linear maxillary tooth row posterior to the sixth maxillary alveolus, (3) a narrow maxilla, not broader than the tooth row between the tooth row and the suborbital fenestra, (4) a surangular-angular suture lingually meeting the articular close to its ventral tip, (5) a lingual foramen for the articular artery perforating only the surangular, and (6) an occlusion pit in line with the tooth row posterior to the 14^th^ dentary alveolus.

*Diplocynodon kochi* Venczel & Codrea, 2022 in having (1) a smooth premaxilla surface lateral to the external naris, (2) a small incisive foramen, (3) a suborbital fenestra reaching anteriorly the level of the eighth or ninth maxillary tooth, (4) an occlusion pit between the seventh and eighth maxillary alveolus with all other dentary teeth occluding lingually, and (5) posterior teeth and alveoli circular in cross-section.

*Diplocynodon muelleri* (Kälin, 1936) in having (1) an external naris opening flush with the dorsal surface of the premaxilla, (2) a narrow maxilla, not broader than the tooth row between the tooth row and the suborbital fenestra, (3) an occlusion pit between the seventh and eighth maxillary alveolus with all other dentary teeth occluding lingually, (4) a suborbital fenestra reaching anteriorly the level of the eighth or ninth maxillary alveolus, (5) the anterior tip of the splenial projecting ventral to the Meckelian groove, (6) a surangular continuing to the dorsal tip of the glenoid fossa, and (7) a large sulcus next to the anterior half of the glenoid fossa.

*Diplocynodon ratelii* Pomel, 1847 in having (1) an occlusion pit between the seventh and eighth maxillary alveolus with all other dentary teeth occluding lingually, (2) a suborbital fenestra reaching anteriorly the level of the eighth or ninth maxillary alveolus, (3) a lingual foramen for the articular artery perforating only the surangular, (4) an occlusion pit in line with the tooth row posterior to the 14^th^ dentary alveolus, and (5) a large sulcus next to the anterior half of the glenoid fossa.

*Diplocynodon remensis* Martin et al., 2014 in having (1) a narrow maxilla, not broader than the tooth row between the tooth row and the suborbital fenestra, (2) a linear maxillary tooth row posterior to the sixth maxillary alveolus, (3) a suborbital fenestra reaching anteriorly the level of the eighth or ninth maxillary alveolus, (4) a splenial excluded from the mandibular symphysis, (5) a shorter mandibular symphysis reaching the level of the third to fourth dentary alveolus, (6) a lingual foramen for the articular artery perforating only the surangular, (7) an occlusion pit in line with the tooth row posterior to the 14^th^ dentary alveolus, (8) a large sulcus next to the anterior half of the glenoid fossa, and (9) posterior teeth and alveoli circular in cross-section.

*Diplocynodon tormis* Buscalioni, Sanz & Casanovas, 1992 in having (1) an occlusion pit between the seventh and eighth maxillary alveolus with all other dentary teeth occluding lingually, (2) an ectopterygoid abutting the last maxillary tooth, and (3) an occlusion pit in line with the tooth row posterior to the 14^th^ dentary alveolus.

*Diplocynodon ungeri* (Prangner, 1845) in having (1) a narrow maxilla, not broader than the tooth row between the tooth row and the suborbital fenestra, (2) a shorter mandibular symphysis only reaching the level of the third to fourth dentary alveolus, (3) a surangular continuing to the dorsal tip of the glenoid fossa, and (4) an occlusion pit in line with the tooth row posterior to the 14^th^ dentary alveolus.

*Diplocynodon buetikonensis* (Meyer, 1854) in having (1) a more elongated retroarticular process, (2) a less notched lateral margin of the glenoid fossa of the articular and (3) a larger third and fourth dentary alveolus compared to the fifth dentary alveolus.

*Diplocynodon* sp. from Romania (Sabău et al., 2021) in having (1) a larger gap between the first and second dentary alveolus, and (2) an occlusion pit in line with the tooth row posterior to the 14^th^ dentary alveolus.

*Diplocynodon* sp. from Ukraine (Kuzmin & Zvonok, 2021) in having a slenderer coracoid shaft.

**Holotype:** No holotype was erected by Huene & Nikoloff (1963), and the described material consists of multiple individuals. The most complete fragment (NMNHS 31-1) is only preserved as a cast, and the original material is missing according to Berg (1966) and Rauhe & Rossmann (1995). They considered this lower jaw to be the holotype. The cast is indeed the most diagnostical bone, as most other fragments lack valuable information. We therefore decided to erect NMNHS 31-1 (depicted in Huene & Nikoloff, 1963 pl. 30, Fig. 1), cast of the right dentary fragment from the first to the 14^th^ alveolus as the lectotype for *D. levantinicum*.

**Type locality and horizon:** All described material is from the Kipra coal-seam of the underground mines Nadeshda (holotype locality) and Radievo 1 of the upper Oligocene West-Maritsa lignite field in the Opan Graben, north from the City of Dimitrovgrad in Central Bulgaria (Figs. 1 and 2).

**Referred material:** The material of *D. levantinicum* consists of at least six different individuals. Most elements are from the lower jaw and postcranial region, whereas only three elements are from the skull. The type series, belonging to at least four individuals previously described by Huene & Nikoloff (1963) (marked with an “H&N”), were found in Summer 1961 by engineer Ivan Nikoloff. They are from the Kipra coal seam from the Nadeshda underground coal mine. Material from at least two additional individuals has been found in Autumn 1981 in the same coal seam from the Radievo 1 underground coal mine.

At least four individuals were assigned to the collection number NMNHS FR 30 and two other individuals to the collection number NMNHS FR 31. NMNHS FR 30 consists of: NMNHS FR 30-1 (left premaxilla fragment), NMNHS FR 30-2 (left dentary fragment), NMNHS FR 30-3 (dentary fragment), NMNHS FR 30-4 (right dentary fragment), NMNHS FR 30-5 (left surangular fragment), NMNHS FR 30-6 (left ventral angular fragment), NMNHS FR 30-7 (left posterior angular fragment), NMNHS FR 30-8 (left articular fragment), NMNHS FR 30-9 (right articular fragment), NMNHS FR 30-10 (cervical vertebra fragment), NMNHS FR 30-11 (caudal vertebra fragment), NMNHS FR 30-12 (right coracoid fragment) (H&N), NMNHS FR 30-13 (right ulna fragment), NMNHS FR 30-14 (metatarsal fragment), NMNHS FR 30-15 (phalange), NMNHS FR 30-16 (dorsal osteoderm) (H&N), NMNHS FR 30-17 (dorsal osteoderm) (H&N), NMNHS FR 30-18 (dorsal osteoderm) (H&N), NMNHS FR 30-19 (ventral osteoderm) (H&N), NMNHS FR 30-20 (ventral osteoderm) (H&N), NMNHS FR 30-21 (left posterior maxilla fragment) (H&N), NMNHS FR 30-22 (left jugal fragment, matching the maxilla fragment) (H&N), NMNHS FR 30-23 (right anterior dentary fragment), NMNHS FR 30-24 (tooth) (H&N), NMNHS FR 30-25 (tooth) (H&N), and NMNHS FR 30-26 (tooth fragment) (H&N).

Note: The left surangular (NMNHS FR 30-7) and left articular (NMNHS FR 30-9) clearly belong to a single individual as the sutures match. Maxilla (NMNHS FR 30-21) and jugal (NMNHS FR 30-22) clearly belong to a single individual as the sutures match. Based on their size, NMNHS FR 30-24, 25 and 26, could belong to a single individual, but are clearly too large to belong to any of the other individuals of NMNHS FR 30.

NMNHS FR 31 consists of NMNHS FR 31-1 (right dentary with 14 alveoli) (cast) (H&N), and NMNHS FR 31-2 (right anterior dentary fragment) (cast) (H&N).

**Preservation:** The material is uncompressed and three-dimensionally preserved, but disarticulated and fragmented.

**Remarks:** Although disarticulated, based on the findings in the same basin and their overall similar size, together with no contradicting morphologies it is reasonable to refer all the found material to the same species, as was previously done by Huene & Nikoloff (1963).

## Description

General shape

*Diplocynodon levantinicum* is a medium to large sized diplocynodontine. The material described here belongs mostly to small to medium sized individuals, with skull lengths between 200 and 250 mm. Disarticulated teeth indicate the presence of considerably larger individuals within the size range of 350 to 400 mm. Based on the estimated skull size, the estimated body size would have lied between 1.75 and 3.00 m (Whitaker & Whitaker, 2008).

Cranial openings (Figs. 3 and 4)

The external naris (Fig. 3A) projects dorsally. Its lateral margin is smooth and rounded, indicating an oval to round shape of the opening. The incisive foramen (Figs. 3A and 3B) is small based on its lateral margin, but it is unclear if it contacts the premaxillary teeth anteriorly. The suborbital fenestra (Fig. 3H) is very long and projects anteriorly to the level of the eighth maxillary alveolus. Its lateral margin is almost straight with only a slight bowing and mainly formed by the maxilla. The external mandibular fenestra is present and shows a clear concavity on its posteroventral margin formed by the angular (Figs. 4D, 4F and 4G).

Premaxilla (Figs. 3A and 3B)

Part of the left premaxilla (NMNHS FR 30-1) is preserved. The lateral surface is weakly ornamented, whereas the inside of the external naris is smooth, with a single small foramen close to the dorsal margin. At the tooth row, small foramina for the receptor canals are exposed. In ventral view, the first four alveoli are partwise preserved, with the fourth one being the largest. Of the latter, the first three alveoli are close to each other whereas a small gap is present between the third and fourth. Medial to the tooth row, small foramina are present.

Maxilla (Figs. 3F–3H)

The posterior part of the left maxilla (NMNHS FR 30-21) is preserved and matches the left jugal (NMNHS FR 30-22) (Figs. 3I–3K). The lateral surface is weakly ornamented, but has well exposed foramina for the receptor canals dorsal to the tooth row, which are strongest between the ninth and 13^th^ alveolus. Posterodorsally, the articulation surface for the jugal is visible. The medial surface is smooth except for the posteriorly opened foramen for the posterior branch of the maxillary nerve, at the level of the 12^th^ alveolus. Posteroventrally, the articulation surface for the ectopterygoid is visible, reaching anteriorly the level of the 13^th^ alveolus. In ventral view, the straight, anteroposteriorly projecting toothrow is visible. On the anteriormost part of the toothrow, an occlusion pit for a dentary tooth (presumably the enlarged 12^th^ tooth) is visible. In *Diplocynodon*, there are usually two occlusion pits present on the maxilla, one posterior to the sixth and one posterior to the seventh alveolus. This indicates that the alveolus posterior to the pit in *D. levantinicum* represents the eighth maxillary alveolus. The total number of teeth is 16. Medial to the tooth row, the maxilla is very slender and bears a row of small foramina. The ectopterygoid is not preserved, but its anterior shape can be identified based on its articulation surface. The ectopterygoid borders the posteriormost alveolus; the penultimate alveolus, is still bordered by the maxilla, but the thin bone part forming its margin is broken off.

Jugal (Figs. 3I–3K)

Part of the left jugal (NMNHS FR 30-22) is preserved and matches the left maxilla (NMNHS FR 30-21) (Figs. 3F–3H). The lateral surface is heavily ornamented with deep pits forming a row ventral to the orbital margin. The medial surface is smooth, but bears some foramina. The large foramen jugularis is exposed lateral to the articulation surface with the ectopterygoid. Anterior to the foramen jugularis, there is another smaller foramen. On the anterodorsal part of the bone fragment, there are two further smaller nutrition foramina present.

Dentary (Figs. 4 and 5)

Six dentary parts are preserved (NMNHS FR 30-2, NMNHS FR 30-3, NMNHS FR 30-4, NMNHS FR 30-23, NMNHS FR 31-1, NMNHS FR 31-2), representing at least four individuals. The ventrolateral surface is weakly ornamented with rounded pits anteriorly and elongated grooves posteriorly. The region directly ventral to the tooth row is overall smooth but foramina for the receptor canals are exposed. The length of the dentary symphysis differs between the individuals. Whereas in NMNHS FR 30-23, NMNHS FR 31-1, NMNHS FR 31-2, the symphysis extends posteriorly only to the level of the third alveolus or to the anterior margin of the fourth one; it reaches the posterior margin of the fourth alveolus in NMNHS FR 30-2. The splenial is excluded from the symphysis and its anterior tip passes ventral to the Meckelian groove. In medial view, the dentary does not possess any foramina, but a canal is exposed projecting from anterodorsal to posteroventral at the level of the 14^th^ alveoli, possibly yielding a nerve or blood vessel in the living animal. The toothrow is gently curved between the fourth and 10^th^ alveoli. The first alveolus is separated from the second by a large gap, and the second from the third by a smaller gap. The third and fourth alveolus are the same size and confluent. The fifth tooth is separated from the fourth by a small gap. The gaps get larger until reaching the ninth alveolus, posterior to which the interalveolar space becomes small again. The 12^th^ alveolus is the largest posterior to the fourth one. Posterior to the 14^th^ alveolus, there is an occlusion pit present which lies in row with the alveoli and broadly separates the 14^th^ from the 15^th^ alveolus. Anteromedial to the tooth row, small foramina are present.

Surangular (Figs. 6A–6C)

Most of the left surangular is preserved (NMNHS FR 30-5). The posterolateral surface is heavily ornamented with deep rounded pits, whereas the anterodorsal surface is smooth. Posterodorsally, the surangular continues to the dorsal tip of the lateral wall of the glenoid fossa and possesses a sulcus lateral to the fossa. In dorsal view, the surangular is uniformly narrow posteriorly, but abruptly broadens anteriorly. On the broaden anterior region, a small foramen is exposed. In medial view, the lingual foramen for the articular artery is exposed entirely on the surangular, indicated by the well visible articulation surface for the articular. Anteriorly, there are two ventrally opening foramina present lying in an anteroposterior projecting row.

Angular (Figs. 6D–6G)

From the left angular, two elements are preserved, a posteroventral fragment (NMNHS FR 30-6) and a posterior fragment (NMNHS FR 30-7). The lateral surface of NMNHS FR 30-6 is heavily ornamented with deep rounded to elongated pits. In medial view, the bone is broken ventrally, but the articulation surfaces for the articular and surangular are well exposed, indicating that the surangular-angular suture meets the articular close to its ventral tip. NMNHS FR 30-7 represents the anteroventral part of the retroarticular process, with an ornamented anterior part and a laterally exposed unornamented posterior part.

Articular (Figs. 6H–6K)

A left (NMNHS FR 30-8) and right (NMNHS FR 30-89) articular fragment are preserved, with the latter being more complete. The glenoid fossa is lateromedially more expanded than anteroposteriorly and medially slightly rounded. The lateral margin is straight anteroposteriorly. The ridge between the glenoid fossa and the retroarticular process is laterally much higher than medially. The foramen aerum is medially shifted and lies at the anterior margin of the ridge. The retroarticular process is medially expanded and tapers posteriorly. The posteriormost expansion of the process lies on the same height as the glenoid fossa. The medial surface of the articular is smooth, without any visible foramen.

Dentition (Figs. 3C–3E)

Two teeth are preserved in the maxilla and multiple teeth or teeth fragments are preserved in the lower jaw fragments. They are conical in shape and have a smooth surface with only slight wrinkles present. Anteriorly and posteriorly, carinae are present. Three further disarticulated teeth are preserved (NMNHS FR 30-24, NMNHS FR 30-25, NMNHS FR 30-26), which, based on their size, belonged to a larger individual, but otherwise show the same morphology as the teeth articulated with the maxilla and lower jaw.

Cervical vertebra (Figs. 7A–7D)

From the cervical vertebra (NMNHS FR 30-10), only the centrum is preserved, but its exact position is unknown. The surface of the centrum is smooth, with a small nutritional foramen present on its ventral half. The hypapophysis is broken off, but expanded about half of the centrum length. The parapophysis projects almost horizontally from the centrum. The posterior condylus is as broad as high.

Caudal vertebra (Figs. 7E and 7F)

From the caudal vertebra, only a posterior fragment (NMNHS FR 30-11) is preserved. The ventral margin of the centrum is slightly concave and the posterior condylus is as broad as high.

Coracoid (Figs. 7G–7I)

Most of the proximal part of the right coracoid (NMNHS FR 30-12) is preserved, whereas the anteriormost part and the blade are broken off. In proximal view, the glenoid is round and slightly posteriorly shifted. Anterior to the glenoid, the articulation surface with the scapula gets gradually narrow. The coracoid foramen lies anterodistal from the glenoid. The shaft of the coracoid is narrow and bowed medially.

Ulna (Figs. 7J–7L)

The proximal articulation surface and part of the shaft of the right Ulna (NMNHS FR 30-13) are preserved. The articulation surface is triangular with a rounded distinct olecranon process. The articulation surface with the radius is straight. The shaft is circular in cross section with a proximodistally projecting ridge.

Metatarsal (Figs. 7M and 7N)

The distal articulation surface and part of the shaft of a metatarsal (NMNHS FR 30-14) are preserved. The distal articulation surface is only slightly broader than the shaft and projects ventrally, whereas the shaft is completely straight.

Phalange (Figs. 7O–7Q)

A single phalange (NMNHS FR 30-15) is preserved. The bone is dumbbell shaped in dorsoventral view and proximally broader than distally in lateral view. The proximal articulation surface is triangular, whereas the distal articulation surface is divided in two condyles.

Osteoderms (Figs. 7R and 7S)

Three dorsal osteoderms (NMNHS FR 30-16, NMNHS FR 30-17, NMNHS FR 30-18) and two ventral osteoderms (NMNHS FR 30-19, NMNHS FR 30-20) are preserved, but their exact position is unknown. Two of the dorsal osteoderms are nearly quadratic, but it is unclear if they are from the midline region. The third osteoderm is slightly elongated and might originate from the dorsolateral region. The outer surface is highly ornamented with deep pits, whereas the inner surface is smooth. A midline keel is present in all dorsal osteoderms. The posterior facet is well developed. The ventral osteoderms are highly ornamented with rounded pits as well, but lack a midline keel. It is unclear if the ventral osteoderms are complete or if they only represent the posterior part of a paired ossification.

## Comparison with other *Diplocynodon* species

Skulls

*Diplocynodon levantinicum* is a medium to large sized *Diplocynodon* species. The largest dentary fragment indicates a skull size of around 250 mm, but isolated teeth belong to a much larger individual with a skull size ranging between 350 to 400 mm. Skull size in Diplocynodontinae varies, some species are relatively large, reaching over 350 mm (*D. elavericus*, *D. ungeri*, *D. hantoniensis*). In contrast, most species lying close to or below 300 mm (*D. kochi*, *D. muelleri*, *D. remensis*, *D. ratelii* and *D. darwini*) and few even below 200 mm *(D. deponiae* and *D. buetikonensis*).

Cranial openings

The cranial openings of *D. levantinicum* are poorly preserved. The external naris is only known from its lateral part, but its margin is well preserved and indicates a dorsal orientation (Fig. 3). This is also true for most other *Diplocynodon* species, except for *D. darwini* in which the naris is opened anterodorsally. The suborbital fenestra in *D. levantinicum* is exceptionally long and reaches anteriorly the level of the eighth maxillary alveolus (Fig. 3) (see discussion below). In *Diplocynodon* the suborbital fenestra is always long, but mostly reaches anteriorly only to the level of the 10^th^ maxillary alveolus (*D. ratelii*, *D. muelleri*, *D. remensis*, *D. hantoniensis*, *D. kochi*) (*e.g.*, Piras & Buscalioni, 2006: fig. 4; Martin et al., 2014: fig. 2; Rio et al., 2020: fig. 2; Venczel & Codrea, 2022: fig. 6). In two taxa (*D. elavericus* and *D. darwini*) the fenestra reaches the ninth alveolus (Berg, 1966: fig. 1; Martin, 2010: fig. 2), whereas only in *D. deponiae* (Delfino & Smith, 2012: fig. 3) does it also reach the eighth alveolus. The condition is unknown for *D. ungeri*, and *D. tormis*.

Skull and lower jaw bones

The premaxilla in *D. levantinicum* is smooth lateral to the external naris (Fig. 3). This is congruent with most *Diplocynodon* species but contrasts with *D. hantoniensis* and *D. kochi* which possess a notch lateral to the naris (Rio et al., 2020: fig. 2; Venczel & Codrea, 2022: fig. 4), as well as in contrast to the premaxilla of *D. muelleri*, in which the naris is circumscribed by a thin crest (Piras & Buscalioni, 2006: fig. 3). In ventral view, the maxilla of *D. levantinicum* is very narrow between the tooth row and the suborbital fenestra anterior to the ectopterygoid (Fig. 3), whereas, in most *Diplocynodon* species, this area is broader than the tooth row (*e.g.*, Martin, 2010: fig. 2; Delfino & Smith, 2012: fig. 3; Rio et al., 2020: fig. 2). A narrow medial part is otherwise only present in *D. ratelii* (Luján et al., 2019: fig. 5), *D. darwini* (Berg, 1966: pl. 1 Fig. 4) and *D. kochi* (Venczel & Codrea, 2022: fig. 6). The maxillary tooth row of *D. levantinicum* has an occlusion pit anterior to the eighth maxillary alveolus (Fig. 3) with all teeth further posterior being laterally positioned to the dentary tooth row. In *D. ratelii*, *D. muelleri*, *D. tormis* and *D. kochi*, on the other hand, occlusion pits posterior to the eighth maxillary alveolus are in line with the maxillary tooth row (*e.g.*, Piras & Buscalioni, 2006: fig. 4). The jugal possesses a large foramen jugularis in *D. levantinicum* (Fig. 3), which is present in most other *Diplocynodon* species as well (*e.g.*, Chroust et al., 2021: fig. 3), but is much smaller in *D. darwini*. The ectopterygoid of *D. levantinicum* abuts the maxillary tooth row for the last maxillary alveolus (Fig. 3); this is also the case for most other *Diplocynodon* species (*e.g.*, Rio et al., 2020: fig. 2), to the exception of *D. deponiae* and *D. tormis* in which the maxilla forms the medial margin of the last alveolus.

The lower jaw symphysis of *D. levantinicum* slightly differs in length between individuals but is always very short. In two individuals (NMNHS FR 30-23, NMNHS FR 31-2) it only reaches the third alveolus, whereas in one individual (NMNHS FR 31-1), it reaches the anterior margin of the fourth alveolus and in another (NMNHS FR 30-2), the posterior margin of the fourth alveolus (Fig. 4). In most *Diplocynodon* species the symphysis is short, reaching not further posterior than the fourth alveolus (*e.g.*, Rio et al., 2020: fig. 10), in *D. darwini* and *D. ungeri*, however, the symphysis is more expanded and reaches at least the fifth dentary alveolus (Martin & Gross, 2011: fig. 6). The lower jaw symphysis in *D. levantinicum* further comprises only the dentary, which is the case in most *Diplocynodon* species, but in *D. remensis* and *D. deponiae* the splenial participates in the symphysis as well (Delfino & Smith, 2012: fig. 3; Martin et al., 2014: fig. 5). In most *Diplocynodon* species in which the splenial does not participate in the symphysis, its anterior tip is situated ventral to the Meckelian groove, as it is the case for *D. levantinicum*. In *D. muelleri*, however, the anterior tip lies dorsal to the Meckelian groove. In *D. levantinicum*, there is an occlusion pit on the dentary tooth row posterior to the 14^th^ alveolus (Figs. 4 and 5), which is in line with the rest of the tooth row. This is unique among *Diplocynodon*, in which the maxillary teeth leave pits either completely lateral to the dentary tooth row, as in *D. remensis* (Martin et al., 2014: fig. 5), or only a slight interfingering is visible as in *D. hantoniensis* (Rio et al., 2020: fig. 9). The surangular possesses a large sulcus lateral to the glenoid fossa in *D. levantinicum* (Fig. 6), which is for *Diplocynodon* otherwise only known for *D. remensis* and *D. hantoniensis* and, outside *Diplocynodon*, only present in *Eoalligator chunyii* Young, 1964 (Wang, Sullivan & Liu, 2016: fig. 6) and some crocodyloids, *e.g., Asiatosuchus germanicus* (Berg, 1966: pl. 4 Fig. 2). The surangular of *D. levantinicum* further continues to the dorsal tip of the lateral wall of the glenoid fossa (Fig. 6), whereas it is truncated in *D. muelleri* and *D. ungeri* (Martin & Gross, 2011: fig. 6). In *D. levantinicum*, the lingual foramen for the articular artery is entirely surrounded by the surangular (Fig. 6), which is among Diplocynodontinae otherwise only reported for *D. muelleri*. The region is, however, not preserved in *D. tormis*, *D. deponiae*, *D. elavericus* and *D. ungeri*. The surangular-angular suture meets the articular at the tip in medial view in *D. levantinicum* (Fig. 6), which is also the case in most other Diplocynodontinae but contrasts with the condition found in *D. hantoniensis*, in which the suture meets the articular dorsal to the tip (Rio et al., 2020: fig. 9).

Teeth

The general tooth morphology of *D. levantinicum* does not differ from the one found in other *Diplocynodon* species, besides that the posterior teeth are rounded in contrast to the laterally compressed teeth in *D. elavericus* (Martin, 2010: fig. 2), *D. remensis* (Martin et al., 2014: fig. 6) and *D. kochi* (Venczel & Codrea, 2022: fig. 6). The number of maxillary teeth, however, differs from most other Diplocynodontinae. *Diplocynodon* usually comprises 17 teeth (*D. elavericus*, *D. remensis*, *D. muelleri*, *D. hantoniensis*, and *D. darwini*) in the maxilla (*e.g.*, Martin, 2010: fig. 2; Martin et al., 2014: fig. 2; Rio et al., 2020: fig. 2), whereas there are only 16 in *D. levantinicum* (Fig. 3) and in *D. deponiae* (Delfino & Smith, 2012: fig. 3) (see discussion below). In *D. kochi* the number of maxillary teeth is not entirely clear. Venczel & Codrea (2022) mention a likelihood of 16 teeth in total, but based on Venczel & Codrea (2022: fig. 6), 17 teeth also seem possible.

Postcranial skeleton

Despite osteoderms, postcranial material of *Diplocynodon* is only well preserved in half of the taxa (*D. ratelii*, *D. hantoniensis*, *D. muelleri*, *D. darwini* and *D. deponiae*). In the coracoid, the coracoid foramen is situated slightly more anterior in *D. levantinicum* (Fig. 7) than in *D. hantoniensis* (Rio et al., 2020: fig. 21). Only two ventral osteoderms are preserved in *D. levantinicum* (Fig. 6). It is likely that the latter represent the posterior part of a bipartite, ossified osteoderm typical for *Diplocynodon*, but no associated anterior part was found.

*Diplocynodon buetikonensis*, *Diplocynodon* sp. from Romania and *Diplocynodon* sp. from Ukraine

*Diplocynodon buetikonensis* is known from the upper freshwater Molasse (Miocene) from Switzerland and Southern Germany (Scherer, 1978; Scherer, 1979; Scheyer, Straehl & Sánchez-Villagra, 2015). Whereas the material from Germany is fragmented and only partially preserved, the anterior part of a skull and lower jaw are known from the Swiss material (Scheyer, Straehl & Sánchez-Villagra, 2015: fig. 40), which, based on its size, could represent a subadult individual. In comparison to *D. levantinicum*, *D. buetikonensis* has a less pronounced curvature of the lower jaw profile. Furthermore, the material from Germany reveals two additional differences. First, in *D. levantinicum*, the fifth alveolus is much smaller than the third and fourth, whereas the size difference is much less severe in *D. buetikonensis* (Scherer, 1979: fig. 1). Second, the lateral margin of the glenoid fossa of the articular is relatively straight in *D. levantinicum*, whereas in *D. buetikonensis* the articular is notched in the middle (Scherer, 1979: fig. 2).

Besides a nearly complete skull recently described as *D. kochi* by Venczel & Codrea (2022), further Romanian *Diplocynodon* material was reported from upper Eocene and lower Oligocene deposits, but not assigned to a certain species (Codrea & Venczel, 2020; Sabău et al., 2021). Although probably belonging to *D. kochi* (Venczel & Codrea, 2022, personal communication), this assignment is not entirely clear, and we therefore decided to address the material separately. The bones comprise of lower jaw fragments, but due to their fragmentary nature, only two distinct differences can be observed. In *D. levantinicum*, the first and second dentary alveolus are separated by a large gap, whereas in *Diplocynodon* sp. from Romania, the two alveoli are close to each other (Sabău et al., 2021: Figs. 2 and 3). The other difference lies in an occlusion pit visible in the lower jaw of *D. levantinicum* between the 14^th^ and 15^th^ dentary alveolus, whereas there is no occlusion pit visible in the material from Romania (Sabău et al., 2021: Figs. 2 and 3). A similarity is, however, seen in the length of the mandibular symphysis. As in *D. levantinicum*, its length lies between the third and fourth dentary alveolus in the Romanian specimens (Codrea & Venczel, 2020: fig. 3; Sabău et al., 2021: Figs. 2 and 3).

*Diplocynodon* material was also reported from the middle Eocene Ikovo locality in Ukraine, but is undiagnostic beyond genus level (Kuzmin & Zvonok, 2021). The latter material comprises of fragments of the skull table, a coracoid and osteoderms. Due to the fragmentary nature of *Diplocynodon* sp. and *D. levantinicum* only the coracoid is preserved in both. In *D. levantinicum* the shaft of the coracoid is narrow, whereas it is very broad in the *Diplocynodon* from Ukraine (Kuzmin & Zvonok, 2021: fig. 6).

## Discussion of the comparison

The maxillary tooth count in *D. levantinicum* is problematic. Most *Diplocynodon* species have 17 maxillary alveoli (*D. elavericus*, *D. remensis*, *D. muelleri*, *D. hantoniensis*, and *D. darwini*), and an occlusion pit between the seventh and eighth alveolus (*D. deponiae*, *D. elavericus*, *D. remensis*, *D. hantoniensis*, and *D. darwini*). If the occlusion pit in the maxilla of NMNHS FR 30-21: Fig. 3H is also situated between the seventh and eighth alveolus, the total number of alveoli is only 16 for *D. levantinicum*, which is otherwise only the case for *D. deponiae* and, presumably, for *D. kochi*. However, tooth count variability is known for *Diplocynodon* and reported for *D. remensis* (Martin et al., 2014), whereas an occlusion pit posterior to the eighth alveolus is only known for *D. ratelii*, *D. tormis* and *D. muelleri*, in which posterior dentary teeth occlude in line with the maxillary toothrow. We therefore conclude that the tooth posterior to the occlusion pit of *D. levantinicum* is indeed the eighth, and that the total number of teeth is 16. Consequently, the size of the suborbital fenestra is longer and projects anteriorly to the level of the eighth maxillary alveolus. This is otherwise only reported for *D. deponiae*, whereas in most *Diplocynodon* species it reaches anteriorly only to the level of the 10^th^ alveolus (*D. ratelii*, *D. muelleri*, *D. remensis*, *D. hantoniensis* and *D. kochi*). The combination of an occlusion pit between the seventh and eighth alveolus, presence of 16 teeth, and a long suborbital fenestra reaching the eighth alveolus, is among Diplocynodontinae otherwise only known from *D. deponiae*. However, the latter has, among other traits, a broader maxilla bar between the toothrow and the fenestra, whereas this bar is narrow in *D. levantinicum*.

Huene & Nikoloff (1963) had already proposed *D. levantinicum* as a valid *Diplocynodon* species, yet based their results solely on the supposed late Pliocene age and geographically distant position of the locality, without comparing it with other *Diplocynodon* species, which would have been necessary for a proper assessment. Although only fragmentarily preserved, the comparison shows that *D. levantinicum* can be distinguished from every other known *Diplocynodon* species, as well as from *Diplocynodon* lower jaw fragments from Romania, by more than a single characteristic, therefore, suggesting that it is indeed a valid species.

## Results of the phylogenetic analyses

**Figure 8:**
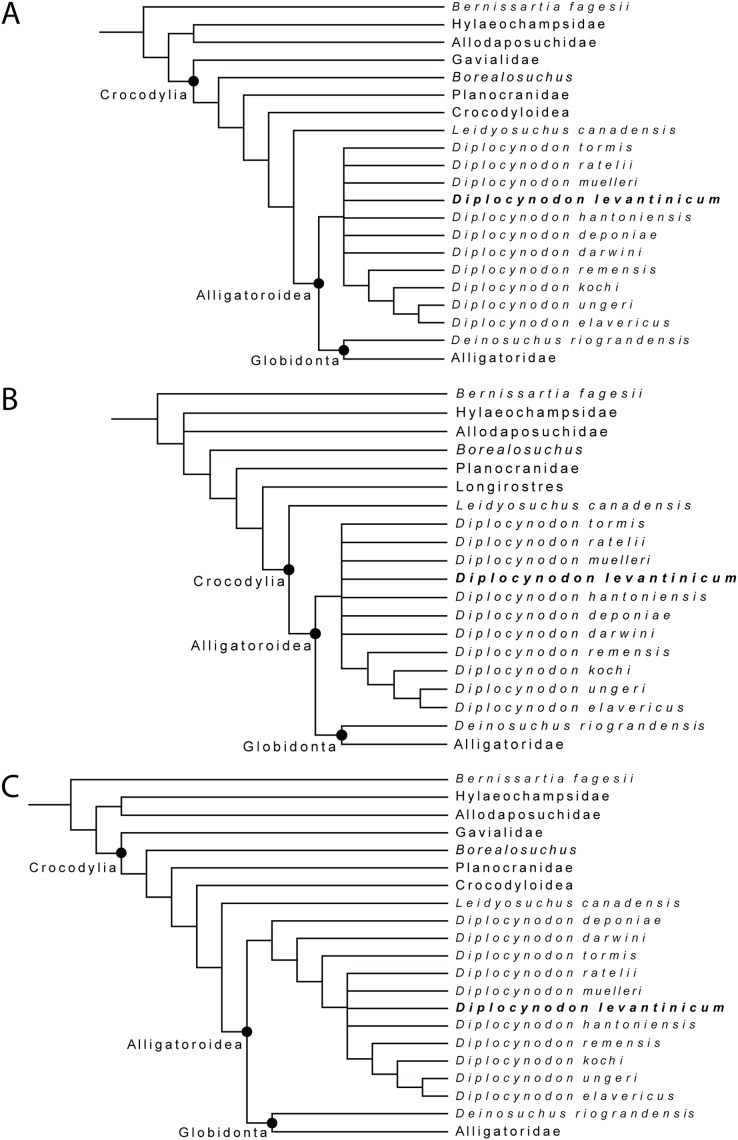
Collapsed Strict consensus trees, obtained from the maximum parsimony analyses of 105 taxa with 187 characters included. (A) Strict consensus tree of 99,999+ equally optimal trees without molecular constrains; length: 763 steps; CI: 0.309 and RI: 0.786. (B) Strict consensus tree of 99,999+ equally optimal trees with applied molecular constrains; length: 775 steps; CI: 0.305 and RI: 0.781. (C) Strict consensus tree of five equally optimal trees without molecular constrains and with extended implied weighting *k* = 20; length: 20,62792 steps; CI: 0.309 and RI: 0.785.

For the first analysis, without a molecular scaffold and without extended character weighting the Traditional Search maximum parsimony analysis yielded a total of 99,999+ equally optimal trees with a length of 763 steps, a consistency index (CI) of 0.309, and a retention index (RI) of 0.786 (Fig. 8A). The same analysis, with a New Technology Search, yielded 877 trees, where the same length, and same consistency and retention indices were found, resulting in no differences between both trees regarding the positioning of taxa. The second analysis, with a molecular scaffold and without extended character weighting, yielded 99,999+ equally optimal trees with a length of 775 steps, a consistency index (CI) of 0.305, and a retention index (RI) of 0.781 (Fig. 8B). The same analysis with a New Technology Search, yielded 1,204 trees with equal length, and equal consistency and retention indices found, resulting in no differences between both trees regarding the positioning of taxa. The third analysis, without a molecular scaffold but with an extended implied weighting of *k* = 20, yielded a total of five equally optimal trees with a length of 20, 62,792 steps, a consistency index (CI) of 0.309, and a retention index (RI) of 0.785 (Fig. 8C). The same analysis, with a New Technology Search, also yielded five trees with the same length, and the same consistency and retention indices, resulting in no differences between both trees regarding the positioning of taxa.

In all analyses, Diplocynodontinae were retrieved as monophyletic and in a basal position inside Alligatoroidea, with *Leidyosuchus canadensis* being the sister taxon to all other alligatoroids, which is congruent with former analyses (*e.g.*, Brochu, 1999; Brochu, 2011; Lee & Yates, 2018; Rio et al., 2020; Shan et al., 2021).

The newly added *D. levantinicum* is always found as deeply nested in a polytomy inside Diplocynodontinae, but the topology of the tree inside the group varies with the parameter used for the analysis. The first analysis without a molecular scaffold and without extended character weighting, and second analysis with a molecular scaffold and without extended character weighting, recover a large polytomy inside Diplocynodontinae. Only the position of *D. remensis*, *D. kochi*, *D. ungeri* and *D. elavericus* are resolved. *D. remensis* is ancestral to the latter three species, and *D. kochi* was found as sister taxon to *D. ungeri* and *D. elavericus*.

The highest resolution inside Diplocynodontinae yields the analysis with an extended implied weighting of *k* = 20. *Diplocynodon deponiae* is found as the basalmost taxon, followed by *D. darwini* and *D. tormis*. *Diplocynodon levantinicum* is found in uncertain relationships with *D. ratelii*, *D. muelleri*, *D. hantoniensis*, and in a monophyletic group consisting of *D. remensis*, *D. kochi*, *D. ungeri* and *D. elavericus*. Using a stronger down weighting of homoplastic characters (*k* < 20) leads to an artefact in which Allodaposuchidae are found as derived members of Diplocynodontinae, whereas there are no changes to the tree topology inside *Diplocynodon* otherwise (see File S2). This result seems unlikely and is not replicable if a lower down weighting of homoplastic characters (*k* ≥ 20) is used. A list of autapomorphies for major groups, as well as the complete trees for all phylogenetic analyses, including the tree from an unordered analysis can be found in the File S2.

## Discussion of the phylogenetic analyses

With equal weighting and without the inclusion of *D. kochi* and *D. levantinicum*, the tree topology is the same as in Rio et al. (2020). If included, *D. kochi* is found in a stable position as sister taxon to *D. ungeri* + *D. elavericus*. The fragmentary material of *D. levantinicum*, however, lead to a larger polytomy with most other *Diplocynodon* species. While using extended implied weighting, another difference inside Diplocynodontinae is the position of *D. deponiae* and *D. darwini*. Whereas in Rio et al. (2020), *D. darwini* is found as the basalmost taxon, in our analysis, *D. deponiae* is ancestral. Reason for this difference in the analysis with extended implied weighting is likely the rescoring of characters (180) and (182) regarding the eye colour and scales, both which are not preserved in *Diplocynodon* (see File S2).

As the polytomy of *D. deponiae* and *D. darwini* in our other analyses already suggests, the basalmost *Diplocynodon* taxon is debatable. In Martin et al. (2014); Macaluso et al. (2019), and Rio et al. (2020), *D. darwini* was found basal, whereas in Brochu (1999); Piras & Buscalioni (2006); Martin (2010); Brochu (2011) and Martin & Gross (2011), *D. deponiae* was found as the basalmost taxon, and in Delfino & Smith (2012) *D. deponiae* was even found towards the crown of Diplocynodontinae.

A potential key to solve this problem could be the characters identifying Diplocynodontinae. In this study, the same four *Diplocynodon* autapomorphies resulted, for all analyses: (1) Axial hypapophysis located toward the centre of centrum (15-0), (2) dorsal margin of the iliac blade rounded with smooth border; posterior tip of the blade very deep (34-4) (3) frontoparietal suture linear between the supratemporal fenestrae (149-1), and (4) a single row of postoccipital osteoderms (181-1). From those autapomorphies, only the rounded dorsal margin of the iliac blade with a very deep tip of the blade is exclusively known for *Diplocynodon* among Eusuchians. The axial hypapophysis located toward the centre of centrum is also known for orientalosuchines, *Crocodylus depressifrons* Blainville, 1855, *Maomingosuchus petrolicus* (Yeh, 1958) and *Toyotamaphimeia machikanensis* (Kobatake et al., 1965). The linear frontoparietal suture between the supratemporal fenestrae is widely spread among crocodilians, and differs even between species of the same genus. Having a single row of postoccipital osteoderms is only preserved in three *Diplocynodon* species, and shared with *Alligator sinensis*, *Paleosuchus palpebrosus* and *Tomistoma schlegelii*, but overall rarely scoreable in Eusuchians. Due to its fragmentary nature, none of those characters is preserved in *D. levantinicum*.

**Figure 9:**
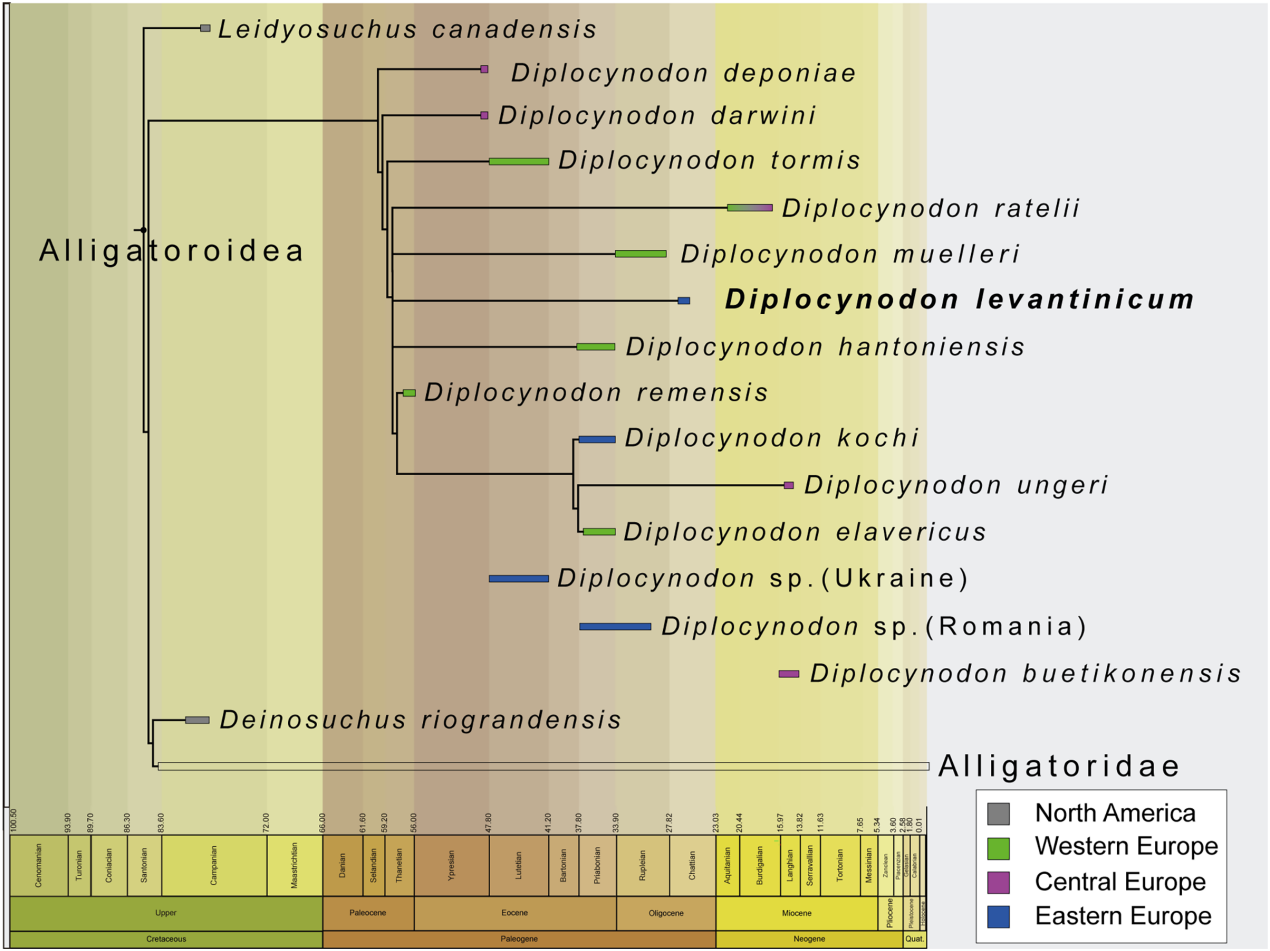
Tine-scaled reduced strict consensus tree with extended implied weighting *k* = 20 of Diplocynodontinae, based on available data from the literature. (modified after Massonne et al., 2021).

The incompleteness of the taxon leads to its unclear relationship with other members of Diplocynodontinae. In the analysis with extended implied weighting, it seems, however, to be closest related to *D. muelleri*, *D. ratelii* and *D. hantoniensis* from Western and Central Europe (Fig. 9). In four out of five trees, *D. levantinicum* forms a sister taxon relationship with *D. muelleri*, which is supported by a single character, as long as *D. ratelii* is found ancestral to this monophylum: lingual foramen for the articular artery and alveolar nerve perforates surangular entirely (69-0), a character state not known for any other *Diplocynodon* species, but also only preserved in half of the taxa. In three out of five trees, there is a single autapomorphy found for *D. levantinicum*: having an occlusion pit between the seventh and eighth maxillary alveolus; whereas all other dentary teeth occlude lingually (92-1), contrasting with *D. ratelii* and *D. muelleri* in which the dentary teeth occlude in line with the maxillary tooth row. However, having an occlusion pit between the seventh and eighth maxillary alveolus is not a rare condition for *Diplocynodon* and can also be found in *D. elavericus*, *D. hantoniensis* and *Diplocynodon remensis*.

The current phylogeny does not support a particular close relationship between *D. levantinicum* from Bulgaria and *D. kochi* from Romania, with *D. kochi* appears to be more closely related to *D. remensis*, *D. ungeri* and *D. elavericus* from Western and Central Europe. Without the usage of extended implied weighting, the close affinity to *D. elavericus* is only based on a single synapomorphy: laterally compressed posterior teeth (79-1). If extended implied weighting is used, the fourth dentary tooth occluding in a notch between the premaxilla and maxilla early in ontogeny (91-0), as well as the postorbital neither contacting the quadrate nor quadratojugal medially (144-0), are found as further synapomorphies. The close relationship of *D. kochi* to *D. ungeri* and *D. elavericus* is in all analyses supported by two synapomorphies: the quadratojugal bearing a modest process, or none at all, along the lower temporal bar (142-1), and a smooth anteromedial corner of the supratemporal fenestra (151-1).

Laterally compressed posterior teeth are reported for *D. remensis*, *D. kochi* and *D. elavericus*, and reversal to rounded teeth reported in *D. ungeri*. The condition is generally uncommon for crocodilians, but does occur in few taxa of Alligatorinae, Caimaninae and Crocodylinae. Among Diplocynodontinae, a notch between the premaxilla and maxilla is only reported for *D. remensis*, *D. kochi* and *D. ungeri*. However, in none of those taxa juvenile specimens seem to be preserved, which opens up the possibility for the presence of a pit given an early ontogenetic state, which is worn away to form a notch later in ontogeny as in some recent caiman species (Brochu, 1999). The postorbital neither contacting the quadrate nor the quadratojugal medially is only reported for *D. remensis*, *D. kochi* and *D. ratelii*. In most *Diplocynodon* taxa, as for *D. elavericus* and *D. ungeri*, this character is, however, unknown, which weakens its significance. Furthermore, *D. kochi*, *D. elavericus* and *D. ungeri* are united by two characters: a quadratojugal bearing only a modest process along the temporal bar, and a smooth anteromedial corner of the supratemporal fenestra, being the only three taxa among Diplocynodontinae, for which this is known.

The problem with a potential closer relationship between *D. kochi* and *D. levantinicum* is the fragmentary nature of the latter. From all the characters supporting a closer relationship of *D. kochi* with *D. remensis*, *D. ungeri*, and *D. elavericus*, only a single one is preserved in *D. levantinicum*. In the Bulgarian taxon, the posterior teeth are rounded, in contrast to *D. kochi*, which is, however, also true for *D. ungeri*. Despite of that, *D. levantinicum* only differs in two other characters from *D. kochi*: a smooth premaxillary surface lateral to the naris (86-0), and an occlusion pit between the seventh and eighth maxillary teeth with all other dentary teeth occluding lingually (92-1). Among Diplocynodontinae, a notch lateral to the external naris is only known for *D. hantoniensis* and *D. kochi*, but looks much smaller in the latter. The different morphology of the notches could be a signal for convergent evolution of this character and does not point towards a particular close relationship between them. In *Diplocynodon*, there is a high amount of variability regarding the occlusion of the tooth rows. In the first group, all dentary teeth occlude lingual to the maxillary teeth (*D. darwini*, *D. deponiae*), in the second group, there is an occlusion pit between the seventh and eighth maxillary alveolus, with all other teeth occluding lingually (*D. elavericus*, *D. hantoniensis*, *D. remensis* and *D. levantinicum*), and in the third group, the dentary teeth occlude in line with the maxillary tooth row (*D. muelleri*, *D. ratelii*, *D. tormis* and *D. kochi*). For *D. ungeri*, the condition is not known. Despite the potential closer relationship between *D. darwini* and *D. deponiae*, there seems to be no phylogenetic pattern for the distribution of those character traits. The relationship between *D. levantinicum* and *D. kochi* with the current material available is thus problematic. Potential characters uniting both taxa, and possibly resulting in a larger group, together with *D. ungeri* and *D. elavericus* are not preserved in *D. levantinicum*, and the characters separating both Eastern European taxa appear to be wide spread in Diplocynodontinae.

We thus conclude that the close affinity of *D. levantinicum* to Western/Central European taxa, instead of a closer affinity to only other known Eastern Europe taxon (*D. kochi*) may be an artefact either produced by the fragmentary nature of *D. levantinicum* and/or due to the absence of more *Diplocynodon* taxa from Eastern Europe. Additional material currently known from Romania and potentially belonging to *D. kochi* (see above) (Codrea & Venczel, 2020; Sabău et al., 2021), and fragments from the Ukraine (Kuzmin & Zvonok, 2021), could be an important factor, but are poorly preserved and have yet to be added to a phylogenetic analysis.

The fragmentary nature of *D. levantinicum* makes its inclusion in a phylogenetic dataset challenging and could proof to be further problematic in the future. In general, the ingroup relationship among *Diplocynodon* taxa seems to be far from solved, demonstrated by the changing relationships between taxa during the last two decades. A general revision of *Diplocynodon* could help in solving these issues and could potentially also help to solve the unclear relationship among basal alligatoroids.

## Conclusion

The validity of *Diplocynodon levantinicum* from the lignite deposits of the West-Maritsa Basin in Central Bulgaria proposed by Huene & Nikoloff (1963) could be verified. *Diplocynodon levantinicum* is characterized by (1) a long suborbital fenestra, (2) a very short dentary symphysis, (3) a large gap between the first and second dentary alveolus, (4) an occlusion pit in line with the tooth row posterior to the 14^th^ dentary alveolus, (5) a sulcus lateral to the glenoid fossa and (6) a lingual foramen for the articular artery situated entirely on the surangular. The current phylogenetic analyses find *D. levantinicum* deeply nested inside Diplocynodontinae in an unresolved polytomy. A closer relationship with *D. kochi* from Romania is currently not supported by the phylogenetic analyses, but because of the fragmentary nature of the material of *D. levantinicum*, better material is certainly needed to test their relationship thoroughly.

The stratigraphic age of the lignite deposits from the West-Maritsa Basin is not late Pliocene, but instead late Oligocene. The Kipra coal seam as the type horizon has an estimated age of ~26 Ma. After the retreat of brackish water Paratethyan influence in the basin, during the early Oligocene (Solenovian stage, Ezerovo Formation), *D. levantinicum* roamed the freshwater lakes and swamps of the Maritsa Formation, contemporary with an intermediate-sized anthracothere and the stem-tragulid “*Dorcatherium*” *bulgaricum*. *Diplocynodon levantinicum* represents the best-documented crocodilian from the late Oligocene in Europe and is the only nominal species of *Diplocynodon* from the Chattian.

## Supplemental Information

10.7717/peerj.14167/supp-1 Supplemental Information 1 Dataset.

10.7717/peerj.14167/supp-2 Supplemental Information 2 Unconstraint dataset.

10.7717/peerj.14167/supp-3 Supplemental Information 3 Molecular constraint dataset.

10.7717/peerj.14167/supp-4 Supplemental Information 4 Unconstraint dataset with implied weighting.

10.7717/peerj.14167/supp-5 Supplemental Information 5 Supplementary 2.

10.7717/peerj.14167/supp-6 Supplemental Information 6 Character list.

## Institutional abbreviations

NMNHS: National Museum of Natural History, Bulgarian Academy of Sciences, Sofia, Bulgaria

## Additional Information and Declarations

### Data Availability

The following information was supplied regarding data availability: Raw data are available in the Supplemental Files.
