# Supplementary material for: Re-evaluation of the morphology and phylogeny of Diplocynodon levantinicum Huene & Nikoloff, 1963 and the stratigraphic age of the West Maritsa coal field (Upper Thrace Basin, Bulgaria)
Source: PeerJ. 2022 Nov 9;10:e14167. doi: 10.7717/peerj.14167 (PMC9653056; doi:10.7717/peerj.14167)
Supplement: Supplemental Information 5 [file peerj-10-14167-s005.pdf]

Supplementary 2

The dataset is identical with the one used in Rio et al. (2020), except for the inclusion of *Diplocynodon levantinicum*. For other taxa, only small adjustments were made (see below):

Diplocynodon darwini

180
Iris (0) greenish/yellowish or (1) brown.
The iris is not preserved. The character will therefore be rescored as (?).

182
Fewer than eight (0) or eight to 14 (1) or more than 14 (2) paired midline scale rows.
The scales are not preserved. The character will therefore be rescored as (?).

Diplocynodon deponiae

180
Iris (0) greenish/yellowish or (1) brown.
The iris is not preserved. The character will therefore be rescored as (?).

182
Fewer than eight (0) or eight to 14 (1) or more than 14 (2) paired midline scale rows.
The scales are not preserved. The character will therefore be rescored as (?).

Diplocynodon elavericus

88
Incisive foramen small, less than half the greatest width of premaxillae (0), or large, more than half the greatest width of premaxillae (1), or large, and intersects premaxillary-maxillary suture (2).

The size of the incisive foramen is not clearly visible in Martin (2010: fig. 2). The character will therefore be rescored as (?)

180
Iris (0) greenish/yellowish or (1) brown.
The iris is not preserved. The character will therefore be rescored as (?).

182
Fewer than eight (0) or eight to 14 (1) or more than 14 (2) paired midline scale rows.
The scales are not preserved. The character will therefore be rescored as (?).

Diplocynodon kochi

97
Preorbital ridges absent or very modest (0) or very prominent (1) at maturity.
There are ridge like structures on the lacrimal visible in Venczel & Codrea (2022: figs. 3, 4), but are more lateral than the classical prefrontal ridges in other taxa. We are unsure if those are homologous to prefrontal ridges in other taxa. The skull part directly anterior to the orbits seems however smooth. The character will therefore be rescored as (0).

102
Maxillary foramen for palatine ramus of cranial nerve V small or not present (0) or very large (1).
The foramen appears rather small in comparison with the large foramen in e.g. *Crocodylus affinis* Brochu (1997: fig. 82). The character will therefore be rescored as (0).

106
Prefrontal dorsal surface smooth adjacent to orbital rim (0) or bearing discrete knoblike processes (1).
There are no knoblike processes visible in Venczel & Codrea (2022: figs. 3, 4). The character will therefore be rescored as (0).

116

Lateral edges of palatines smooth anteriorly (0) or with lateral process projecting from palatines into suborbital fenestrae (1).

There is a slight lateral flaring of the anterior part of the palatine, but after the comparison with Brochu (1997: fig. 92) and other *Diplocynodon* taxa, we would rather interpret the structure in *D. kochi* as smooth. The character will therefore be rescored as (0).

117

Palatine-pterygoid suture nearly at (0) or far from (1) posterior angle of suborbital fenestra.

The palatine-pterygoid suture is not preserved in *D. kochi*. The character will therefore be rescored as (?).

118

Pterygoid ramus of ectopterygoid straight, posterolateral margin of suborbital fenestra linear (0) or ramus bowed, posterolateral margin of fenestra concave (1).

The posterior part of the suborbital fenestra is too badly preserved to score with confidence. The character will therefore be rescored as (?).

119

Lateral edges of palatines parallel posteriorly (0) or flare posteriorly, producing shelf (1).

The posterior part of the palatines is too badly preserved to score with confidence. The character will therefore be rescored as (?).

162

Extensive exposure of prootic on external braincase wall (0) or prootic largely obscured by quadrate and laterosphenoid externally (1).

The prootic seems to be too badly preserved to score with confidence. The character will therefore be rescored as (?).

Diplocynodon remensis

79

Teeth and alveoli of maxilla and/or dentary circular in cross-section (0), or posterior teeth laterally compressed (1), or all teeth compressed (2).

The posterior alveoli visible in Martin et al. (2014: fig. 6) show a mediolateral compression. The character will therefore be rescored as (1).

List of specimens, rescoring was based on:

Diplocynodon darwini: HLMD Me 4281, HLMD Me 5648, HLMD Me 10496

Diplocynodon deponiae: IRSNB R 261, IRSNB R 262, SMF Me 899

Diplocynodon elavericus: Rhinopolis B3

Diplocynodon kochi: UBB V.1453

Diplocynodon remensis: MNHN BR 2636, MNHN BR 13207

Institutional Abbreviations:

HLMD: Hessisches Landesmuseum Darmstadt, Germany;

IRNSB: Institut royal des Sciences naturelles de Belgique, Brussels, Belgium;

MNHN-BR: Muséum National d'Histoire Naturelle – Berru collection, Mourras quarry of Mont de Berru, Paris, France

Rhinopolis-Association: Rhinopolis, Gannat, Allier, France;

SMF: Senckenberg Museum, Frankfurt, Germany;

UBB: Babeș-Bolyai University, Cluj-Napoca, Romania

List of autapomorphies for major clades:

Autapomorphies for molecular constrainted dataset

Crocodylia: 18 (0), **27** (1), **33** (1), **147** (2)

Longirostres: 102 (1), **114** (1), **148** (1), **179** (3)

Alligatoroidea: 61 (1), **70** (1), **72** (1), **103** (1), 175

Diplocynodontinae: 15 (0), **34** (4), **149** (1), **181** (1)

***D. remensis* + *D. kochi* + *D. ungeri* + *D. elavericus*: 79** (1)

***D. kochi* + *D. ungeri* + *D. elavericus*: 142** (1), **151** (0)

***D. ungeri* + *D. elavericus*: 114** (1), **115** (1), **146** (2), **157** (1)

Autapomorphies for unconstrainted dataset

Crocodylia: 63 (1), **149** (0), **156** (1), **172** (1)

Brevirostres: 18 (0), **27** (1), **33** (1), **118** (1), **147** (2)

Crocodyloidea: 102 (1), **114** (1), **148** (1), **179** (3)

Alligatoroidea: 47 (0), **61** (1), **70** (1), **103** (1), **175** (1)

Diplocynodontinae: 15 (0), **34** (4), **149** (1), **181** (1)

***D. remensis* + *D. kochi* + *D. ungeri* + *D. elavericus*: 79** (1)

***D. kochi* + *D. ungeri* + *D. elavericus*: 142** (1), **151** (0)

***D. ungeri* + *D. elavericus*: 114** (1), **115** (1), **146** (2), **157** (1)

Autapomorphies for unconstrainted dataset with implied weighting *k* = 20

Crocodylia: 63 (1), **149** (0), **156** (1) **172** (1)

Brevirostres: 18 (0), **27** (1), **33** (1), **118** (1), **147** (2)

Crocodyloidea: 3 (1), **12** (0), **73** (1), **148** (1), **160** (1), **179** (3)

Alligatoroidea: 47 (0), **61** (1), **70** (1), **103** (1), **139** (1), **175** (1)

Diplocynodontinae: 15 (0), **34** (4), **149** (1), **181** (1)

***D. darwini* + *D. tormis* + *D. muelleri* + *D. levantinicum* + *D. ratelii* + *D. hantoniensis* + *D. remensis* + *D. kochi* + *D. ungeri* + *D. elavericus*: 54** (1)

***D. tormis* + *D. muelleri* + *D. levantinicum* + *D. ratelii* + *D. hantoniensis* + *D. remensis* + *D. kochi* + *D. ungeri* + *D. elavericus*: 52** (1), **92** (2), **101** (1)

***D. remensis* + *D. kochi* + *D. ungeri* + *D. elavericus*: 79** (1), **91** (0), **144** (0)

***D. kochi* + *D. ungeri* + *D. elavericus*: 142** (1), **151** (0)

***D. ungeri* + *D. elavericus*: 114** (1), **115** (1), **146** (2), **157** (1)

Complete strict consensus tree without molecular constraints

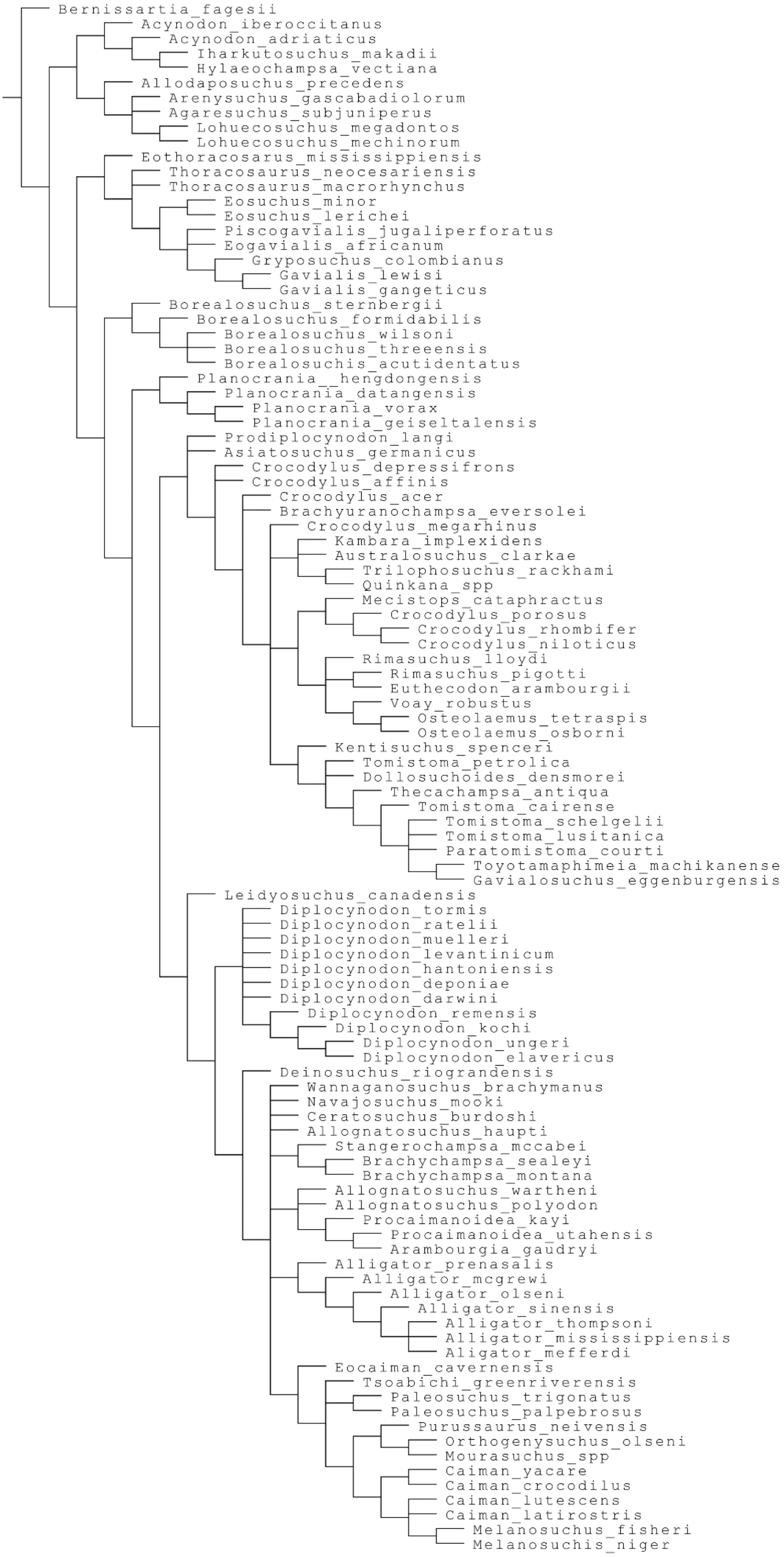

Complete strict consensus tree with applied molecular constraints

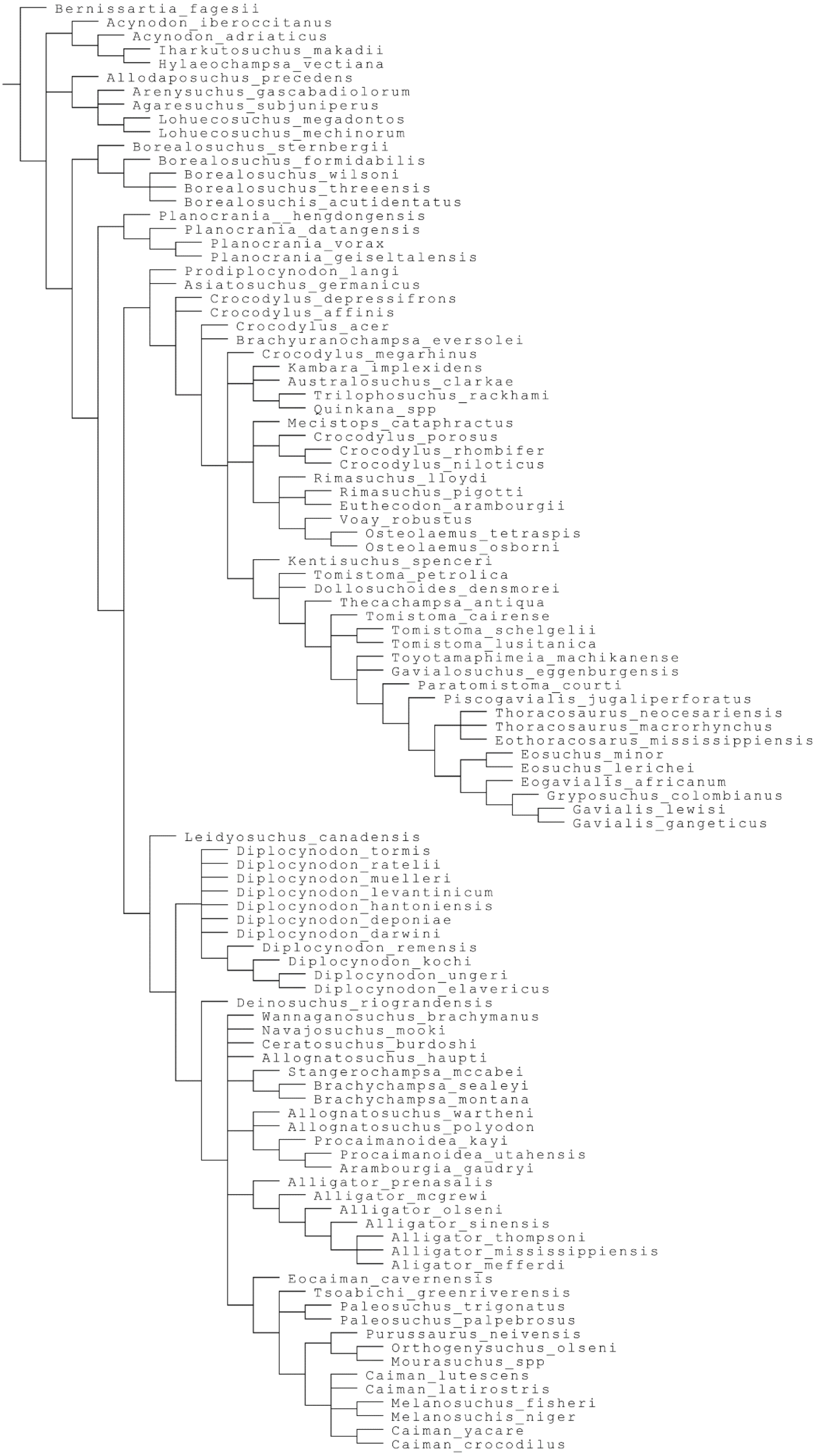

Complete strict consensus tree without molecular constraints and with implied weighting *k* = 20

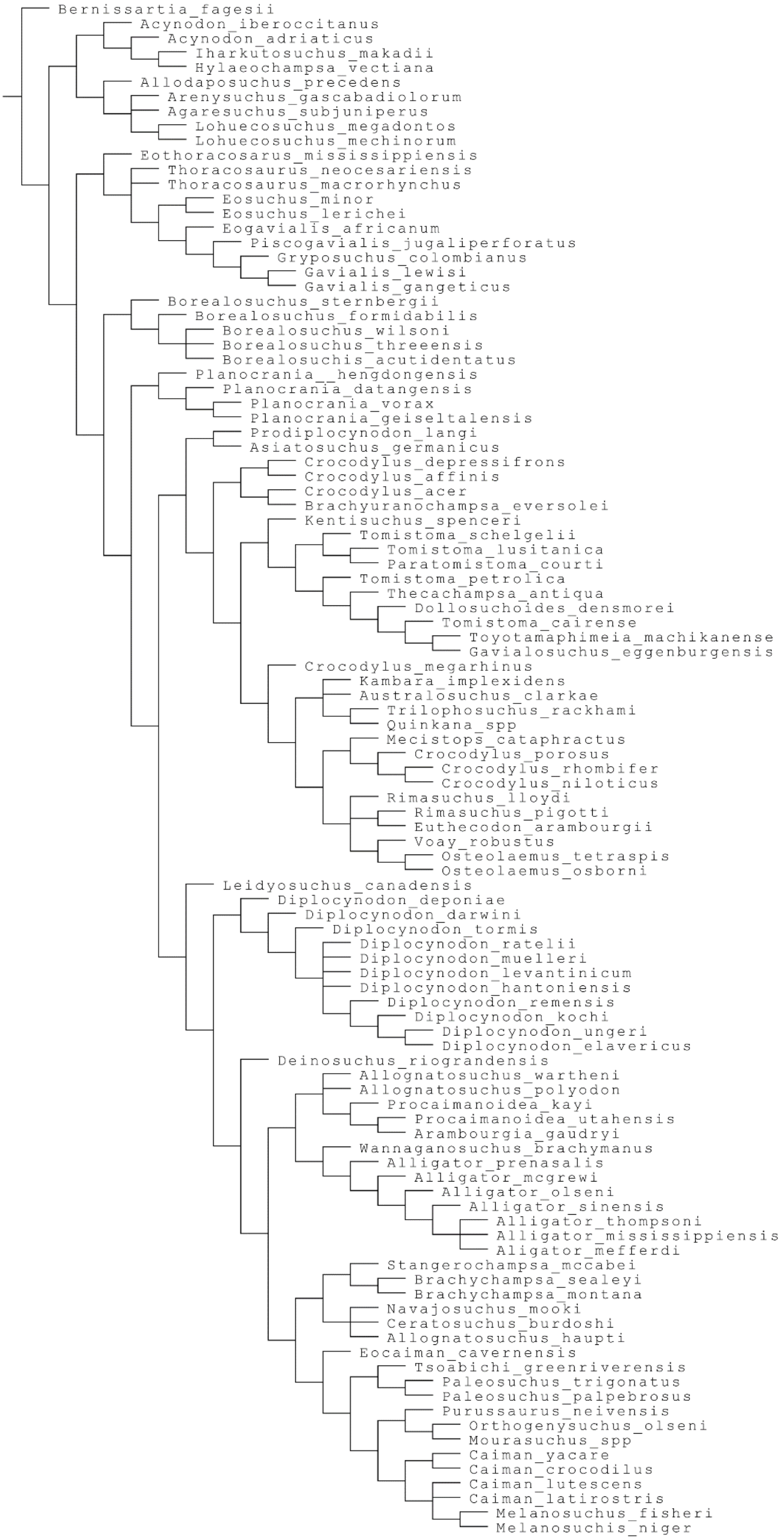

Complete strict consensus tree without molecular constraints and with implied weighting *k* < 20

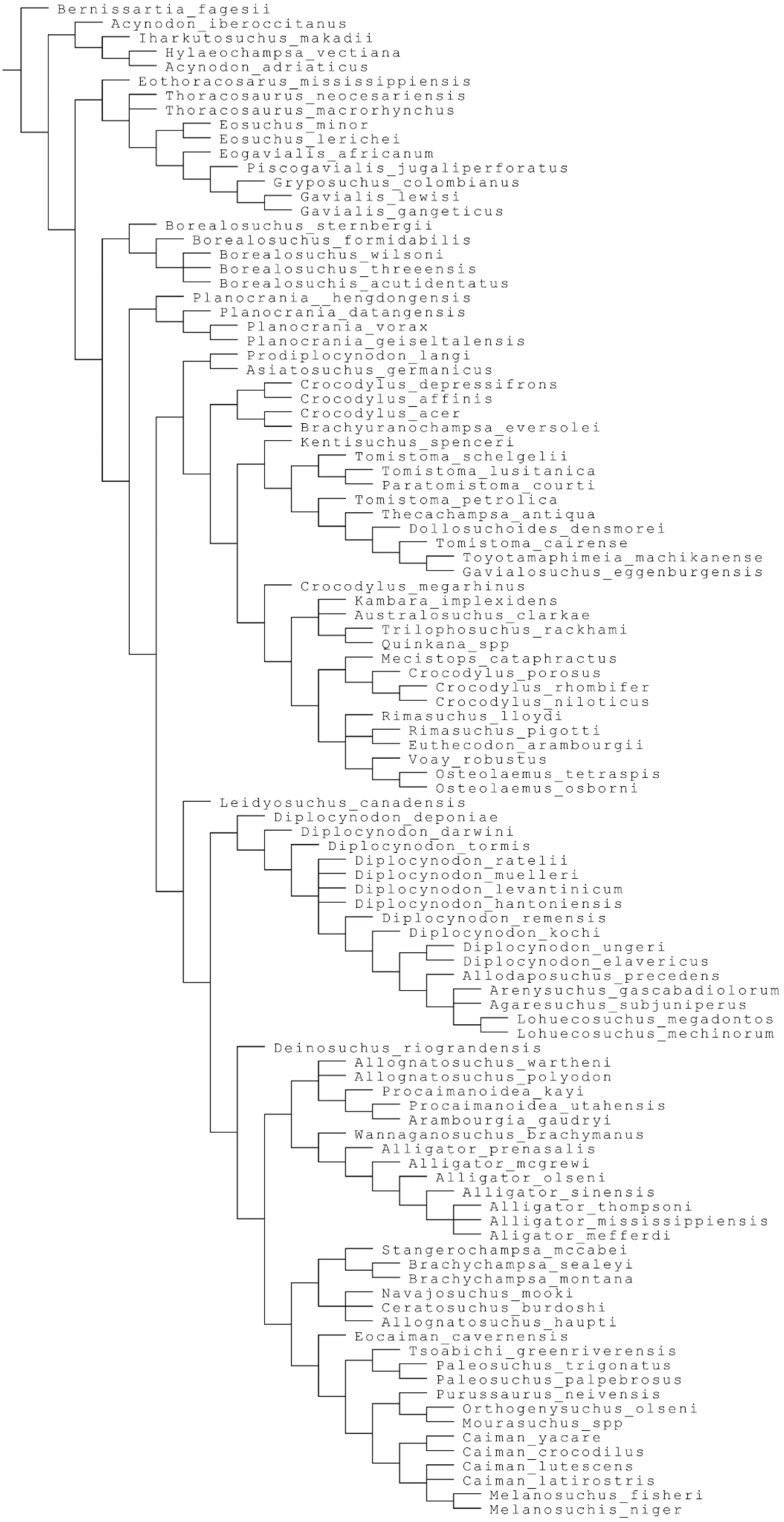

Complete strict consensus tree without ordering, without molecular constraints and without implied weighting *k<20*

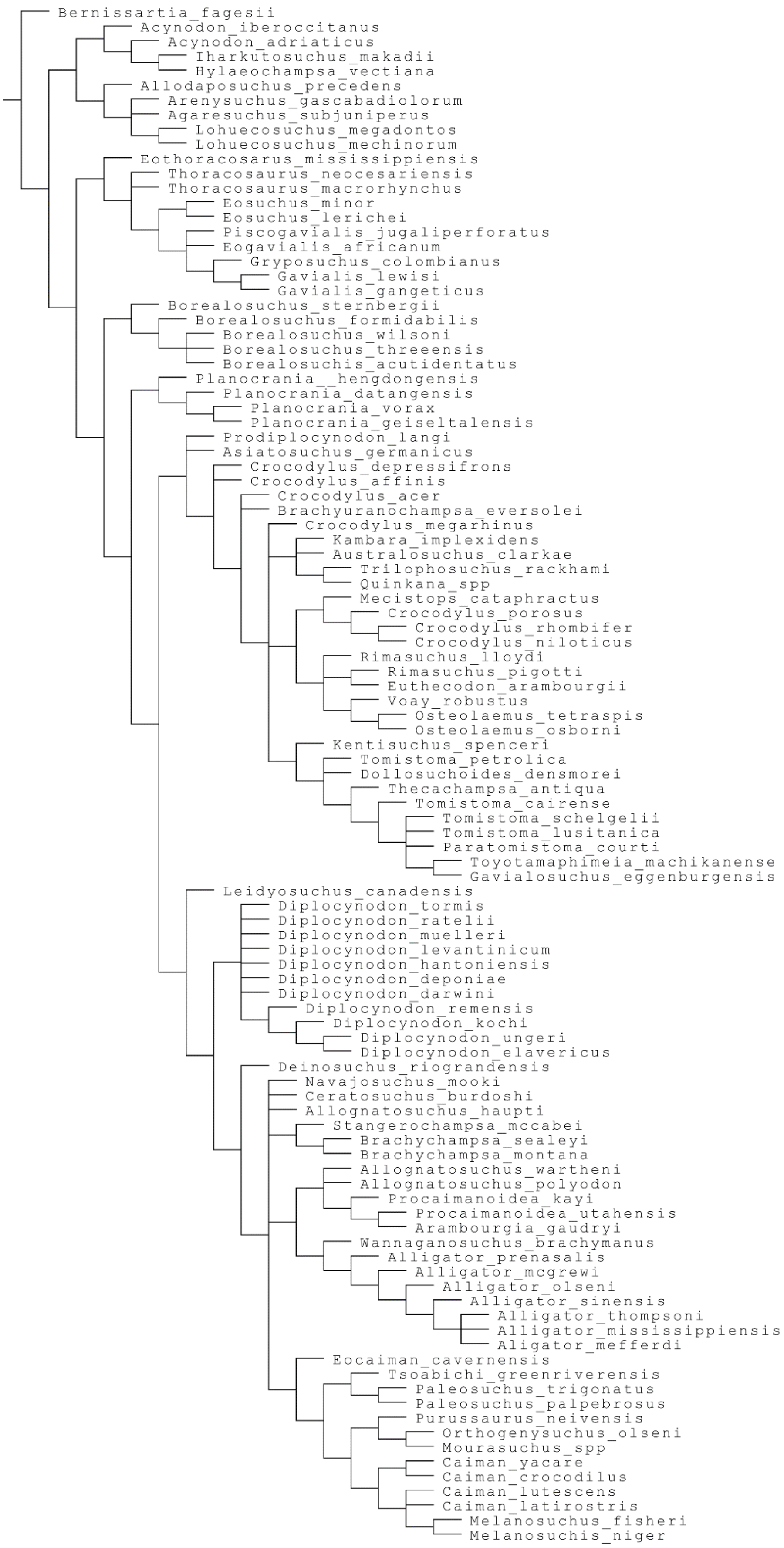

References

Brochu C. A. (1997). Phylogenetic Systematics and Taxonomy of Crocodylia. *Thesis,* Univ. Texas, Austin.

Martin, J. E. (2010). A new species of *Diplocynodon* Crocodylia, Alligatoroidea) from the late Eocene of the Massif Central, France, and the evolution of the genus in the climatic context of the Late Palaeogene. *Geological Magazine 147*(4), 596–610.

Martin, J. E., Smith, T., de Lapparent de Broin, F., Escuillié, F., & Delfino, M. (2014). Late Palaeocene eusuchian remains from Mont de Berru, France, and the origin of the alligatoroid *Diplocynodon*. *Zoological Journal of the Linnean Society*, *172*(4), 867–891.

Rio, J. P., Mannion, P. D., Tschopp, E., Martin, J. E., & Delfino, M. (2020). Reappraisal of the morphology and phylogenetic relationships of the alligatoroid crocodylian *Diplocynodon hantoniensis* from the late Eocene of the United Kingdom. *Zoological Journal of the Linnean Society 188*(2), 579–629.

Venczel, M., & Codrea, V. A. (2022). A new late Eocene alligatoroid crocodyliform from Transylvania. *Comptes Rendus Palevol*, *21*(20), 411–429.
